# Exercise Therapy Versus Manual Therapy for the Management of Pain Intensity, Disability, and Physical Function in People With Chronic Low Back Pain: A Systematic Review With Meta‐Analysis and Meta‐Regression

**DOI:** 10.1002/ejp.70090

**Published:** 2025-08-01

**Authors:** Luis González‐Gómez, Jose A. Moral‐Munoz, Abel Rosales‐Tristancho, Alejandro Cuevas‐Moreno, Melania Cardellat‐González, Álvaro‐José Rodríguez‐Domínguez

**Affiliations:** ^1^ Department of Nursing and Physiotherapy University of Cadiz Cádiz Spain; ^2^ Biomedical Research and Innovation Institute of Cadiz (INiBICA), University of Cadiz Cádiz Spain; ^3^ Department of Statistics and Operational Research University of Seville Seville Spain; ^4^ Department of Health and Sports University Pablo de Olavide Seville Spain

**Keywords:** chronic low back pain, disability, exercise therapy, manual therapy, musculoskeletal pain

## Abstract

**Background and Objective:**

Guidelines recommend combining physical and psychological programmes for chronic low back pain (CLBP); however, exercise therapy (ET) and manual therapy (MT) are often delivered separately. This systematic review with meta‐analysis and meta‐regression of randomised controlled trials (RCTs) aimed to compare the efficacy of ET with MT in terms of pain intensity, disability and physical function in people with CLBP.

**Databases and Data Treatment:**

MEDLINE, Web of Science, PEDro, Cochrane Library and Scopus were searched July–August 2024 for RCTs comparing ET with MT in participants aged 18–54 years. Outcomes were extracted for the short‐, medium‐ and long‐term follow‐up periods. Risk of bias (RoB 2.0 Cochrane Tool) and certainty of evidence (GRADE) were appraised.

**Results:**

Six RCTs (743 patients) were included. Meta‐analyses showed, albeit non‐clinically relevant, a significant difference for long‐term in favour of ET for disability (SMD = −0.25, 95% CI [−0.43, −0.07], *p* = 0.007). Meta‐regression showed that the female–male ratio, treatment duration and mean age explain variability in pain intensity and disability.

**Conclusions:**

ET had a small beneficial effect on long‐term disability in people with CLBP. Nevertheless, evidence does not provide conclusive differences between both the treatments overall, influenced by heterogeneity and the number of studies included. Biopsychosocial factors may moderate the differences in outcomes. The GRADE assessment revealed very low certainty across all outcomes, highlighting the lack of high‐quality research.

**Significance Statement:**

ET may offer small long‐term benefits over MT for disability in people with CLBP. Differences seem to be influenced by sex, age and treatment duration. The choice of ET over MT, or vice versa, as a stand‐alone treatment does not appear to be supported by current evidence.

**PROSPERO Registration Number:**

CRD42024569120

## 1 Introduction

Low back pain (LBP) is a condition with a point prevalence of approximately 12% (Manchikanti et al. 2014), affecting 70%–90% of the general population at some time in their lives (Soriano et al. 2018) and tends to chronification and disability in 2%–48% (Meucci et al. 2015). According to data from the 2017 National Health Survey, the prevalence of LBP in Spain is 22% (Dueñas et al. 2020), and chronic low back pain (CLBP) is more frequent in women with low socioeconomic status and low physical activity (Moreno‐Ligero et al. 2023). The occupational and personal burden of CLBP requires awareness of the available treatments for its management.

Concerning treatment, the National Institute for Health and Care Excellence (NICE) (NICE 2020) on LBP and sciatica advises referring patients to combined physical and psychological programs (CPPP) that encompass supervised exercise therapy (ET), cognitive‐behavioural techniques, self‐management education and training in work‐related activities. Likewise, the National Low Back and Radicular Pain Pathway (NICE 2017) designates CPPP as a core treatment. Nevertheless, ET and manual therapy (MT) are often used unimodally for the management of CLBP, sometimes because of a lack of time or resources, or insufficient academic training in CPPP (Etheridge et al. 2022; Moniz et al. 2024).

Therefore, knowing the effects of one modality over another could be important because it could help optimise resources and improve clinical decision‐making. ET and MT have been proposed as potential noninvasive interventions for the management of CLBP, albeit with limited evidence of low‐ to moderate‐quality (Furlan et al. 2015; George et al. 2021; Hayden, Ellis, Ogilvie, et al. 2021; Qaseem et al. 2017).

Regarding ET, evidence suggests its potential benefits for people with CLBP (Hayden, Ellis, Ogilvie, et al. 2021; Li et al. 2023; Owen et al. 2020). Exercise‐induced hypoalgesia (EIH) has been identified as a potential mechanism for endogenous pain modulation (Kuithan et al. 2019; Tomschi et al. 2025), possibly influenced by opioids, endocannabinoids, non‐opioids and other physiological systems (Rice et al. 2019). Nevertheless, some studies have provided initial evidence of alterations in the mechanisms of EIH in people with chronic pain, which requires further research to establish their relevance to both experimental and clinical pain in people with musculoskeletal pain (Wewege and Jones 2021). In this sense, the biological mechanisms underlying the hypoalgesic effects of exercise are not yet fully understood (Fiuza‐Luces et al. 2013; Vaegter and Jones 2020).

The concept of passive MT in physiotherapy practice encompasses several techniques performed by qualified therapists, including joint and soft tissue mobilisation and manipulation, soft tissue massage and nerve mobilisation (Hidalgo et al. 2014), as defined by the International Federation of Manual and Musculoskeletal Physical Therapists (IFOMPT) (Silvernail et al. 2024). From another perspective, MT is widely accepted as a therapeutic approach in certain branches of clinical practice, such as osteopathy and chiropractic, which constitute autonomous professions with differentiated conceptual, training, regulatory frameworks and safety profiles (Draper‐Rodi et al. 2024; Kerry et al. 2024; Shivachev and Mancheva 2022).

Although various mechanisms of action have been suggested for MT, there is currently no unified theory explaining its underlying neurobiology (Bialosky et al. 2018). Historically, the effects of MT have been attributed to biomechanical changes and specific tissue response. However, emerging evidence suggests that these hypotheses could now be supplemented by neurovascular, endocrine and immunological responses, which are consistent with the nonspecific response attributed to placebo and contextual factors (Keter et al. 2025).

In this regard, a previous review (Gomes‐Neto et al. 2017) compared the efficacy of different ET and MT modalities for the management of pain intensity, disability and function in CLBP. However, it introduced two studies in which patients with sub‐acute LBP were randomised (Rasmussen‐Barr et al. 2003) and a Back School programme was prescribed within the MT protocol (Goldby et al. 2006). These studies did not meet our inclusion criteria, focusing our study on CLBP. Therefore, to our knowledge, this is the first systematic review with meta‐analysis and meta‐regression to compare ET and MT isolated in patients with CLBP.

Given this background, this study aimed to compare the efficacy of ET versus MT in people with CLBP through a systematic review with meta‐analysis and meta‐regression of RCTs. Efficacy was evaluated on the basis of pre‐ and post‐intervention differences in pain intensity, disability and physical function outcomes.

## 2 Methods

### 2.1 Protocol and Registration

A systematic review with meta‐analysis was reported on the basis of the PRISMA (Preferred Reporting Items for Systematic Reviews and Meta‐Analyses) 2020 statement (Ardern et al. 2022). A literature search was conducted in databases between July 1 and August 1, 2024. The protocol was registered with PROSPERO (registration number: CRD42024569120).

### 2.2 Search Strategy

Research questions were formulated using the PICOS (Participants, Interventions, Comparison, Outcomes, Study) approach (Methley et al. 2014): Is ET more effective than MT in reducing pain intensity, improving function and reducing disability in adults with CLBP?

The databases consulted for the identification of studies were MEDLINE, Cochrane Central Register of Controlled Trials (CENTRAL), Web of Science, Scopus and Physiotherapy Evidence Database (PEDro). Studies were not filtered by publication date or language.

The inclusion criteria were as follows: (i) RCTs; (ii) adults aged 18–65 years with CLBP (duration greater than 12 weeks); (iii) ET as the intervention; (iv) MT as the comparison and (v) results related to pain intensity, disability and physical function.

The exclusion criteria were as follows: (i) studies on patients with cancer, pregnant women, acute LBP, and other diseases and (ii) studies whose interventions were combined or did not compare MT and ET alone.

### 2.3 Qualitative Synthesis

Two authors (L.G.‐G., A.‐J.R.‐D.) independently selected studies for review. After removing duplicate studies, filtering by title and abstract and checking compliance with the inclusion criteria, the articles were obtained and examined in their full text. In the analysis, relevant data from each study were independently extracted, and a third author (J.A.M.‐M.) helped in the agreement and resolution of different review processes. The following data were presented in a homogenised scheme, which included information for the qualitative synthesis: authors, year of publication, number and age of participants, interventions performed, study outcomes and their measurement instruments, follow‐up duration and treatment results.

### 2.4 Quality Assessment and Strength of the Evidence

The risk of bias was independently assessed by two authors (L.G.‐G., M.C.‐G.) with the assistance of a third author (J.A.M.‐M.) in the case of disagreement. The objective was to identify selection bias, performance bias, attrition bias, detection bias and selective outcome reporting bias, following the risk of bias criteria recommended by the Cochrane Handbook for Systematic Reviews of Interventions (Cochrane Collaboration 2020). The Review Manager 5.4.1 software (The Cochrane Collaboration, The Nordic Cochrane Centre, Copenhagen, Denmark) was used.

The strength of evidence was assessed using the Grading of Recommendations Assessment, Development, and Evaluation (GRADE) using the GRADE Pro/Guideline Development Tool (Balshem et al. 2011), facilitating the assessment of possible sources of bias, inconsistency, indirectness, imprecision and publication bias.

### 2.5 Quantitative Synthesis

#### 2.5.1 Meta‐Analysis

Two authors (L.G.‐G., A.‐J.R.‐D.) used Review Manager version 5.4.1 to perform the meta‐analysis. A third author (J.A.M.‐M.) resolved any disagreements. Effect sizes and standard errors were calculated for each study. Inverse‐variance methods were applied for weighting in the meta‐analysis. The standardised mean difference (SMD) was determined following the example of one of the studies evaluated (Ferreira et al. 2010), and its standard deviation was calculated using Hedges' adjusted g formula. SMD was considered as the magnitude effect, establishing cut‐off points of 0.20, 0.50, and 0.80, which can be considered to represent a small, moderate and large effect, respectively (Murad et al. 2019). The corresponding 95% confidence intervals are indicated, with statistical significance set at *p* < 0.05. The chi‐square test and *I* ^2^ statistics were employed to assess heterogeneity, with *I* ^2^ values in the range of 0%–40% classified as low, 30%–60% as moderate, 50%–90% as substantial and 75%–100% as considerable heterogeneity. A random‐effects model was used for studies with heterogeneity or fixed effects for studies with homogeneity. We stratified by different follow‐up intervals to provide a broad view of the effects of interventions ranging from 0 to 3 months, 3 to 6 months, or more than 6 months. To avoid double‐counting participants, we appropriately split the control group sample size when the studies compared two different ET modalities (Cochrane Collaboration 2020). A forest plot was created, with ET and MT as the experimental and control groups, respectively. A sensitivity analysis was performed using the Review Manager 5.4.1 software. Each study was successively excluded to assess the impact of its design on the overall measurement of the effect and on the temporal subgroups of the interventions.

In view of the heterogeneity of MT interventions, we decided to perform a secondary analysis excluding Bronfort et al. (2011) and de Oliveira Meirelles et al. (2020), since osteopathy and chiropractic are not techniques but complete professions with their own diagnostic models that transcend MT. When feasible, meta‐analyses and sensitivity analyses were conducted, excluding those studies in accordance with the established procedures above.

#### 2.5.2 Minimum Clinical Difference

To compare our results with the minimum clinically important difference (MCID), only pain intensity and physical function were considered in the meta‐analysis, given the use of different tools to measure disability. A difference of 15% was established for pain intensity (Ferreira et al. 2013) and 10% for physical function (Hayden, Ellis, Ogilvie, Malmivaara, et al. 2021). Owing to the nature of the continuous data, the mean difference (MD) was used as the effect measure because the same measurement scale was applied. Similarly, a secondary analysis was performed, if possible, excluding the aforementioned studies (Bronfort et al. 2011; de Oliveira Meirelles et al. 2020).

#### 2.5.3 Meta‐Regression

Finally, one author (A.R.‐T.) performed a meta‐regression of pain intensity and disability outcomes to explain their effects in terms of other outcomes. The model fit was evaluated through the *R* ^2^ coefficient, and the significance of the explanatory variables was determined from the *p*‐value obtained in each case (taking as reference the significance levels of 1%, 5%, or 10%) (Ipiña and Durand 2008). Considering a correlation coefficient of 0.7 (Cochrane Collaboration 2020), the difference between the pre‐intervention and post‐intervention states was calculated for each comparison and its standard deviation, taking the value of 0–3 months, as this was the period with the most data for the analysis. To reflect the SMD between a single ET modality and MT, the differences previously calculated between the two ET modalities were combined (Cochrane Collaboration 2020). Similarly, the combined data for the independent outcomes that presented two different values were calculated for each exercise value. Finally, the SMD between the above values was calculated for the ET and MT groups, ensuring a homogeneous measure that could be combined in the meta‐regression analysis. It was not possible to estimate meta‐regression models for the independent variables when Bronfort et al. (2011) and de Oliveira Meirelles et al. (2020) were excluded.

### 2.6 Equity, Diversity and Inclusion Statement

The sample of studies analysed in our systematic review included both men and women with CLBP across different age ranges, allowing us to examine the impact of ET and MT while considering outcomes such as sex and age. We acknowledge most of the RCTs included in our analysis were conducted in high‐ and middle‐income countries, which may limit the generalisability of our findings to lower‐resource settings. Additionally, our manuscript discusses the role of biopsychosocial factors, including potential gender biases, in the perception and treatment of CLBP.

## 3 Results

**FIGURE 1:**
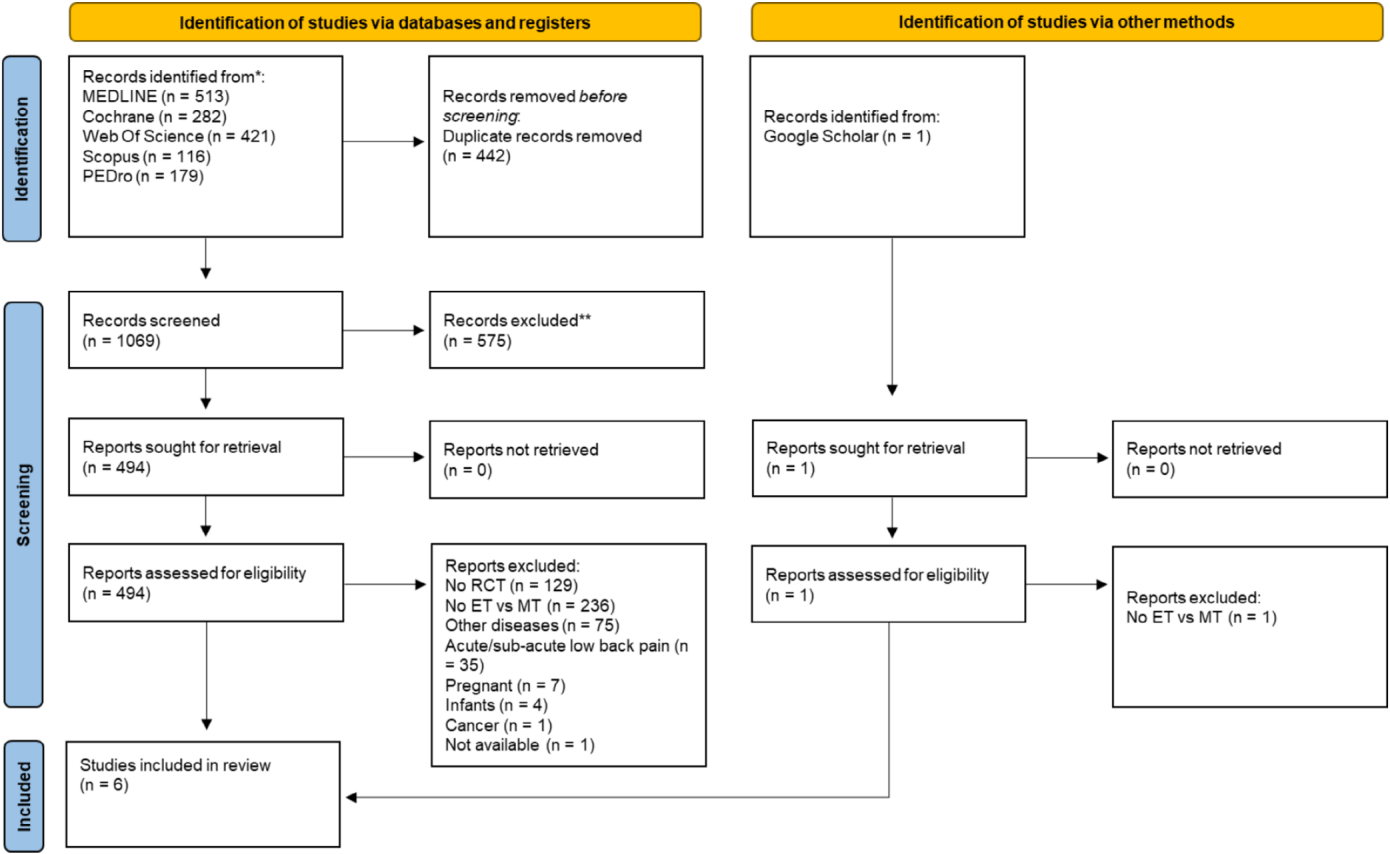
Information flow diagram of the different processes in the systematic review.

The initial search retrieved a total of 1510 studies, of which six (Bronfort et al. 2011; de Oliveira Meirelles et al. 2020; Ferreira et al. 2010, 2007; Ferreira 2009; Ulger et al. 2017; Zhang et al. 2022) were included in both the qualitative synthesis and meta‐analyses. All studies were published in English between 2007 and 2022. The PICOS criteria, MeSH terms and search strings for the different databases are shown in Supporting Information (Table S1). A flowchart is shown in Figure 1. The studies are presented in Table 1 and classified according to the type of intervention performed and the characteristics of their application.

**TABLE 1:** Classification of studies according to the type of intervention.

Type of therapy	Therapy modality	Number of studies	Dosing parameters	Intervention
Exercise therapy	General exercise	4 (Bronfort et al. 2011; de Oliveira Meirelles et al. 2020; Ferreira et al. 2010; Ferreira et al. 2007)	2–3 sets of 15–30 reps Maximum number of repetitions possible in 1 min No more detailed information	Aerobic warm‐up exercises, muscle stretching, strength exercises for the main muscle groups: trunk and leg extensions, abdominal crunches, oblique abdominals, gluteal bridge and side bridge, squats or push‐ups
	Motor control	3 (Ferreira et al. 2010; Ferreira et al. 2007; Ulger et al. 2017)	No more detailed information	Static and dynamic stabilisation of the spine: hollowing, gluteal bridge, side bridge, bear crawl, one‐legged balance, superman, wall squat, dead bug, breathing and pelvic floor exercises
Manual therapy	Mobilisation and massage	6 (Bronfort et al. 2011; de Oliveira Meirelles et al. 2020; Ferreira et al. 2010; Ferreira et al. 2013; Ulger et al. 2017; Zhang et al. 2022)	For post‐isometric relaxation, gentle contraction for 8 s up to 30% of maximum voluntary contraction	Joint mobilisation or manipulation with thrust/non‐thrust techniques, flexion distraction therapy, myofascial release techniques and/or muscle‐energetic techniques related to lumbar region or pelvis
	Osteopathy	1 (de Oliveira Meirelles et al. 2020)	Osteopathic treatment practice. No more detailed information	
	Chiropractic	1 (Bronfort et al. 2011)	Low amplitude and high velocity. No more detailed information	

As shown in Table 2, the sample size of the included studies ranged from 17 to 301 subjects, with a total of 743 patients, of whom 465 were women and 278 were men. The ages of the participants ranged from 28 to 55 years.

**TABLE 2:** Characteristics of the included studies.

Study	Sample	Treatment arms	Groups	Age	Female: male	PEDro score	Intervention	Outcome measure and follow‐up	Reported results
Ferreira et al. (2010)	34 subjects with CLBP	MCE GE SMT	*n* = 11–11 *n* = 10–10 *n* = 13–13	47.5 ± 17.3 54.9 ± 11.3 45.4 ± 17.7	6:5 7:3 10:3	7/11	MCE: training for the function of specific deep muscles of the lumbar region, posture and breathing exercises GE: aerobic warm‐up, stretching and strength training of major muscle groups, cool down and relaxation SMT: joint mobilisation techniques without thrust manipulation to the spine or pelvis Duration: 12 sessions over 8 weeks	(1) NPRS (2) RM Follow‐up at 0 and 8 weeks	All groups improved in the clinical outcomes at the 8‐week follow‐up
Ferreira et al. (2007)	240 subjects with CLBP	GE MCE SMT	*n* = 80–73 *n* = 80–65 *n* = 80–73	54.8 ± 15.3 51.9 ± 15.3 54 ± 14.4	56:24 53:27 56:24	8/11	GE: aerobic warm‐up, stretching and strength training of major muscle groups, cool down and relaxation MCE: TrA, multifidus and pelvic floor muscles training in gradually more functional positions SMT: joint manipulation/mobilisation techniques for spine or pelvis Duration: 12 sessions over 8 weeks	(1) VAS (2) RM Follow‐up at 0, 6 and 12 months	Greater improvement for MCE and SMT in the short term, with no differences in subsequent follow‐ups
Zhang et al. (2022)	17 subjects with CLBP	TE MT	*n* = 9–9 *n* = 8–8	28.11 ± 7.45 28.75 ± 7.26	0:9 0:8	6/11	TE: motor control exercises (trunk and limb muscle stretching, trunk and hip rotation and flexion) and stabilisation exercises (bridge exercise with progression, side bridge exercise, superman, bear crawl exercise and dead bug exercise) for 30′ MT: muscle relaxation, myofascial release, and mobilisation for 20′. Duration: six sessions, once every other day	(1) VAS Measurement at 12 days. No follow‐up	Statistically significant improvements in VAS, with greater effects in the MT group
de Oliveira Meirelles et al. (2020)	42 subjects with CLBP	ACG OMTG	*n* = 19–18 *n* = 23–20	50.1 ± 9.3 46 ± 10.4	12:6 16:4	8/11	ACG: therapeutic exercises not specified. OMTG: osteopathic treatment practice, articulation and myofascial techniques not specified. Duration: 10 sessions twice a week (ACG) and a 5 sessions once a week (OMTG) for 5 weeks	(1) VAS (2) ODI Follow‐up at 0 and 5 weeks	Both groups improved VAS and ODI, with better effects in the MT group
Bronfort et al. (2011)	301 subjects with CLBP	SET HEA SMT	*n* = 100–82 *n* = 101–81 *n* = 100–82	44.5 ± 11.8 45.6 ± 10.3 45.2 ± 10.8	57:43 59:41 66:34	9/11	SET: 2–3 sets of 15–30 repetitions of dynamic core strengthening exercises (back and leg extensions, sit‐ups on the floor and on a gym ball); light aerobic warm‐up; static lumbar and glute stretching; and pre/post‐strengthening stretches for 1 h HEA: simple stretching and strengthening exercises, such as lumbar extension, bridges and sit‐ups, and was encouraged to perform them at home every day SMT: short lever manipulation, low amplitude and high velocity and soft tissue massage during 15–30′ Duration: twice (SET), once/twice (SMT) or daily (HEA) for 12 weeks	(1) VAS (2) RM (3) SF‐36 (physical component) Follow‐up at 0 and 12, 26 and 52 weeks	All groups improved outcomes in the short and long term. Without statistically significant differences in treatment effects
Ulger et al. (2017)	144 subjects with CLBP	SSE MT	*n* = 72–56 *n* = 72–57	43.1 ± 14.3 41.6 ± 12.9	32:24 35:22	6/11	SSE: basic and advanced level exercises, including diaphragmatic breathing and co‐contraction of the TrA and core muscles MT: joint mobilisation and manipulation, soft tissue mobilizations, myofascial stretching for superficial and deep muscles, transverse friction for interspinous and supraspinous ligaments, musculo‐energetic techniques and post‐isometric relaxations for the quadratus lumborum and piriformis muscles Duration: 18 1‐h sessions, three times a week for 6 weeks	(1) VAS (2) SF‐36 (3) ODI Follow‐up 0 at 6 weeks	Both groups improved significantly after treatment, with significant ODI in the MT group

Abbreviations: ACG, active control group; AET, active exercise therapy; CLBP, chronic low back pain; ET, exercise therapy; GE, general exercises; GPE, global perceived effect; HEA, home exercise and advice; MCE, motor control exercises; MT, manual therapy; ODI, oswestry disability index; OMTG, osteopathic manipulation treatment group; PCS, catastrophic pain thinking; PSFS, patient‐specific functional status; RM, Roland‐Morris; SET, supervised exercise therapy; sfMpQ, McGill pain questionnaire; SMT, spinal manipulative therapy; SSE, spinal stabilisation exercises; TE, therapeutic exercise; TrA, transversus abdominis; VAS, visual analogue scale.

The studies examined in this review compared different modalities of ET and MT, with two (de Oliveira Meirelles et al. 2020; Ulger et al. 2017; Zhang et al. 2022) or three (Bronfort et al. 2011; Ferreira et al. 2010, 2007) treatment arms. The targeted outcomes of this review were pain intensity, monitored using the Visual Analog Scale (VAS) (Chiarotto et al. 2019) and the Numeric Pain Rating Scale (NPRS) (Nugent et al. 2021); disability, measured using the Roland‐Morris (RM) questionnaire and the Oswestry Disability Index (ODI) (Jenks et al. 2022); and physical function, collected by the Physical Component Summary (PCS) of the Medical Outcomes Study Short Form 36 (SF‐36) (Gandek et al. 2004).

The intervention programmes had a duration of 5–24 sessions at an interval of 2–12 weeks, with a weekly frequency of 1–7 sessions, and a follow‐up of 2–52 weeks. In some studies, the duration of the MT session ranged 20–30 min (Bronfort et al. 2011; Zhang et al. 2022), whereas the ET session lasted from 10 min to 1 h (Bronfort et al. 2011; Ulger et al. 2017; Zhang et al. 2022). Other studies did not specify the treatment duration (Ulger et al. 2017). Two studies replicated the same exercise program (Ferreira et al. 2010, 2007), and one did not specify the ET protocol (de Oliveira Meirelles et al. 2020). One study included arms with two exercise protocols within its treatment: one supervised and the other with home instructions (Bronfort et al. 2011).

Regarding the risk of bias assessment of the studies included in this review (Figures S1 and S2), the overall risk of bias was high.

**FIGURE 2:**
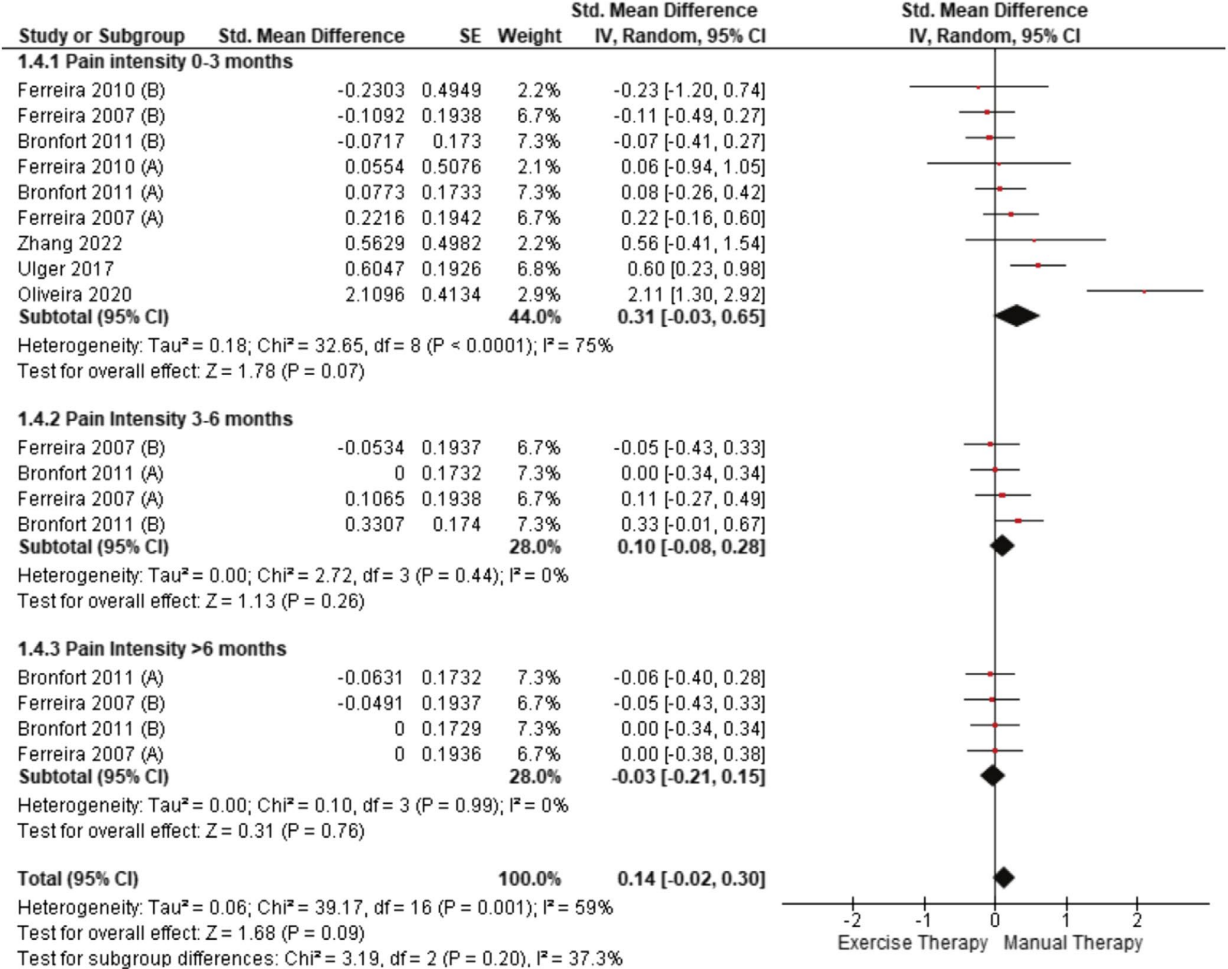
Comparison between ET and MT for pain intensity in CLBP (forest plot of the meta‐analysis).

The meta‐analyses performed for each outcome were divided into subgroups, classified according to the temporality of the post‐intervention measurements, with stratification at 0–3 months, 3–6 months or more than 6 months. Five studies (Bronfort et al. 2011; de Oliveira Meirelles et al. 2020; Ferreira et al. 2007; Ulger et al. 2017; Zhang et al. 2022) assessed pain intensity using the VAS, whereas another study (Ferreira et al. 2010) used the NPRS (Figure 2). No statistically significant differences were found between the ET and MT groups at follow‐up at 0–3 months (SMD = 0.31; 95% CI [−0.03, 0.65], *p* = 0.07), 3–6 months (SMD = 0.10; 95% CI [−0.08, 0.28], *p* = 0.26), more than 6 months (SMD = −0.03; 95% CI [−0.21, 0.15], *p* = 0.76), or overall (SMD = 0.14; 95% CI [−0.02, 0.30], *p* = 0.09). When a secondary analysis was performed excluding Bronfort et al. (2011) and de Oliveira Meirelles et al. (2020) (Figure S3), statistically significant differences with small effect in favour of MT were observed for the 0–3 months period (SMD = 0.23; 95% CI [0.02, 0.43], *p* = 0.03) with a decrease in heterogeneity between studies (*I* ^2^ = 59% to 20%).

**FIGURE 3:**
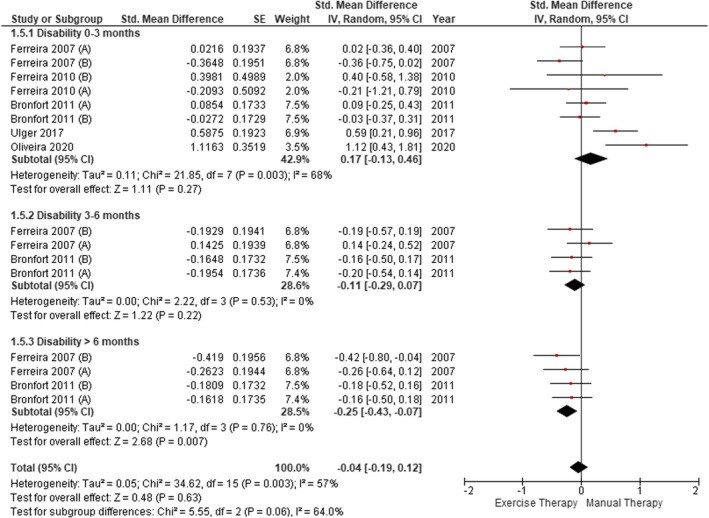
Comparison between ET and MT for disability in CLBP (forest plot of the meta‐analysis).

Five of the six studies (Bronfort et al. 2011; de Oliveira Meirelles et al. 2020; Ferreira et al. 2010; Ferreira et al. 2007; Ulger et al. 2017) incorporated disability assessment, two (de Oliveira Meirelles et al. 2020; Ulger et al. 2017) used the ODI scale and three (Bronfort et al. 2011; Ferreira et al. 2010; Ferreira et al. 2007) used the RM questionnaire (Figure 3). No statistically significant differences were found between the two groups at 0–3 months (SMD = 0.17; 95% CI [−0.13, 0.46], *p* = 0.27) and 3–6 months (SMD = −0.11; 95% CI [−0.29, 0.07], *p* = 0.22). There were statistically significant differences with a small effect in favour of ET for more than 6 months (SMD = −0.25; 95% CI [−0.07, −0.43], *p* = 0.007). No statistically significant differences were found between ET and MT in the overall measurement (SMD = −0.04; 95% CI [−0.19, 0.12], *p* = 0.63). Excluding Bronfort et al. (2011) and de Oliveira Meirelles et al. (2020) (Figure S4), statistically significant differences with a small effect in favour of ET remained for more than 6 months (SMD = −0.34; 95% CI [−0.61, −0.07], *p* = 0.01), with a similar heterogeneity between studies (*I* ^2^ = 57% to 62%).

**FIGURE 4:**
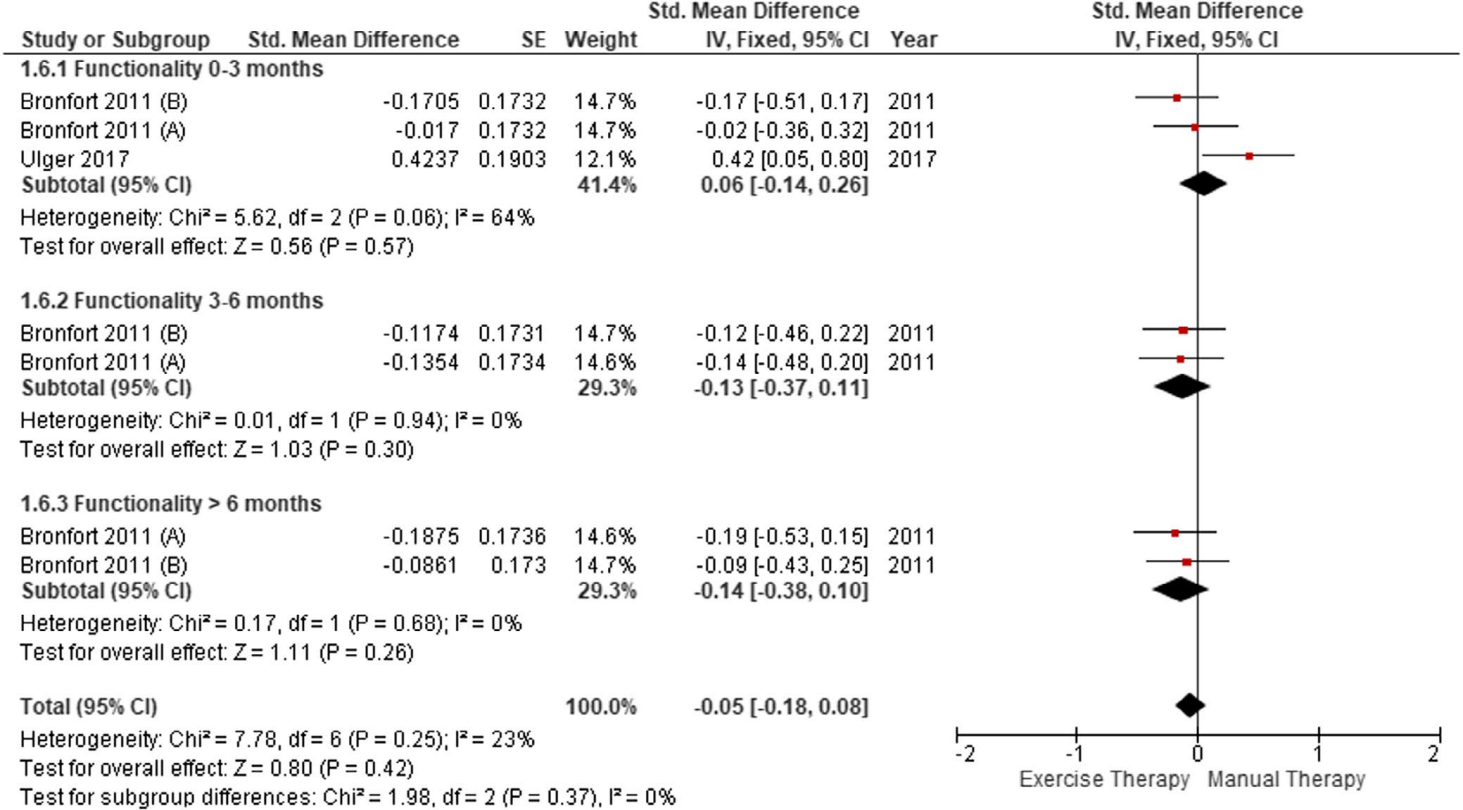
Comparison between ET and MT for physical function in CLBP (forest plot of the meta‐analysis).

Two of the six studies included in this review (Bronfort et al. 2011; Ulger et al. 2017) examined physical function using the SF‐36 tool (Figure 4). No statistically significant differences were found between ET and MT at the follow‐up of 0–3 months (SMD = 0.06; 95% CI [−0.14, 0.26], *p* = 0.57), 3–6 months (SMD = −0.13; 95% CI [−0.37, 0.11], *p* = 0.30) and over 6 months (SMD = −0.14; 95% CI [−0.38, 0.10], *p* = 0.26). In the global analysis, there were no statistically significant differences between the ET and MT groups (SMD = −0.05; 95% CI [−0.18, 0.08], *p* = 0.42). It was not possible to perform the meta‐analysis excluding Bronfort et al. (2011) and de Oliveira Meirelles et al. (2020) because of a lack of sufficient data.

The strength of the evidence was evaluated using the GRADEproGDT tool, obtaining ‘very low’ evidence for the three outcomes studied, the results of which were classified as ‘not important’ (Table S2). Sensitivity analysis (Tables S3–S10), with and without the studies by Bronfort et al. (2011) and de Oliveira Meirelles et al. (2020), showed that excluding any study did not significantly affect the direction of the effect at any follow‐up or overall when considering SMD as the magnitude of effect. Regarding the MCID for CLBP, it was possible to compare the results obtained for pain intensity on the basis of the dimensionality shared by the VAS and NPRS tools and their physical function. Meta‐analyses showed no clinically relevant improvement for pain intensity (0.27%) (Figure S5) and physical function (0.57%) (Figure S7). No statistically significant differences were found between the ET and MT groups at the follow‐up of 0–3 months (MD = 0.55; 95% CI [−0.06, 1.15], *p* = 0.08), 3–6 months (MD = 0.19; 95% CI [−0.10, 0.48], *p* = 0.19) or more than 6 months (MD = −0.05; 95% CI [−0.32, 0.22], *p* = 0.72). The overall measurement showed no statistically significant difference between ET and MT (MD = 0.27; 95% CI [−0.01, 0.56], *p* = 0.06). When Bronfort et al. (2011) and de Oliveira Meirelles et al. (2020) were excluded (Figure S6), statistically significant differences were found for pain intensity in favour of MT at the temporality of 0–3 months (MD = 0.42; 95% CI [0.08, 0.77], *p* = 0.02), but not overall (MD = 0.22; 95% CI [−0.03, 0.47], *p* = 0.08). For physical function, no statistically significant differences were found between ET and MT at the temporality of 0–3 months (MD = [0.41; 95% CI [−1.91, 2.73], *p* = 0.73), 3–6 months (MD = −0.75; 95% CI [−1.90, 0.40], *p* = 0.20) and at more than 6 months (MD = −0.80; 95% CI [−1.93, 0.34], *p* = 0.17). In the global analysis, the difference between ET and MT was not statistically significant (MD = −0.57; 95% CI [−1.30, 0.17], *p* = 0.13). Sensitivity analysis revealed that the exclusion of Ulger et al. (2017) (Table S7) affected the effect size, direction (MD = −0.59; 95% CI [−1.24, 0.06]; *p* = 0.29) to (MD = −0.70; 95% CI [−1.36, −0.04]; *p* = 0.04)] and heterogeneity (*I* ^2^ = 18% to 0%), resulting in a statistically significant, albeit non‐clinically relevant, improvement in favour of ET overall in terms of physical function. No analysis on the comparison of physical function with the MCID could be performed when Bronfort et al. (2011) and de Oliveira Meirelles et al. (2020) were excluded.

Meta‐regression was carried out for the difference pre‐post intervention between ET and MT for pain intensity and disability outcomes because of the lack of data to create a model considering the physical function outcome (Table 3).

**TABLE 3:** Meta‐regression for pain intensity and disability.

Variable	Coefficient	SE	*t*‐statistic	95% CI	*p*
Pain					
Constant	−5.5931	1.8150	−3.0816	(−13.4023, 2.2162)	0.0911***
Mean sessions	0.0079	0.0520	0.1522	(−0.2156, 0.2314)	0.8930
Ratio female/male	−1.8017	0.5807	−3.1026	(−4.3003, 0.6969)	0.0901***
Mean age	0.1798	0.0600	2.9980	(−0.0782, 0.4377)	0.0956***
Disability					
Constant	−17.9517	2.6573	−6.7556	(−51.7162, 15.8127)	0.0936***
Mean sessions	0.5042	0.0815	6.1898	(−0.5308, 1.5391)	0.1020
Intervention weeks	0.4264	0.0565	7.5455	(−0.2916, 1.1443)	0.0839***
Ratio female/male	3.9396	0.5934	6.6396	(−3.5997, 11.4790)	0.0952***

Abbreviations: CI, confidence interval; SE, standard error.

* *p* < 0.01.

** *p* < 0.05.

*** *p* < 0.10.

Regarding pain intensity, the meta‐regression model had a value of *R* ^2^ = 0.8010, indicating a good fit. The female/male ratio showed a statistically significant negative effect on the SMD between the ET and MT groups (at a significance level of 10%). The mean age also had a statistically significant but positive effect on the SMD between the two groups (at a significance level of 10%).

In the case of the disability variable, the meta‐regression model had a coefficient of *R* ^2^ = 1. This value would indicate a perfect fit of the model. Nevertheless, this may not be due to a perfect fit but rather to the model having the same number of observations as the parameters to be estimated. Both the female/male ratio and the number of weeks of intervention showed statistically significant positive effects on the SMD between the ET and MT groups (at a significance level of 10%).

## 4 Discussion

To the best of our knowledge, this is the first systematic review with meta‐analysis and meta‐regression comparing the isolated efficacy of ET versus MT in people with CLBP. For this purpose, we concluded that there is insufficient evidence to state that there are global differences between ET and MT in the management of pain intensity, disability and physical function.

Regarding pain intensity, a reduction was observed at all time points and overall, with no statistically significant or clinically relevant differences between the ET and MT groups. These findings are consistent with those of two Cochrane reviews (Hayden, Ellis, Ogilvie, Malmivaara, et al. 2021; Saragiotto et al. 2016). Hayden et al. included studies comparing different ET modalities with MT and showed no statistically significant differences in the first six to 12 weeks for pain intensity. The results obtained in the mid‐term and long‐term follow‐up periods were combined in the meta‐analysis along with other conservative treatments, making the interpretation of the results difficult. Saragiotto et al. reported moderate‐ to high‐quality evidence that a comparison between motor control exercises and MT offers similar results overall. In contrast to these findings, there are clinical practice guidelines and cost‐effectiveness studies that show ‘strong evidence’ in favour of ET in CLPB compared to MT, which still lacks consistent evidence for its isolated use (Corp et al. 2020; Kaito et al. 2019; Wayne et al. 2019). Most studies on the management of CLBP have been conducted in adult populations (Boekel et al. 2022; Fullen et al. 2022; Gouteron et al. 2022). In this regard, meta‐regression analysis suggested that for every one‐unit increase in the mean age of the study population, the improvement in the ET group was 0.17 points greater than that in the MT group for pain intensity. This is consistent with other studies (Bastiaens et al. 2024), which showed that adults obtain better results than young people because chronic pain can be expected in old age and is better managed (Pericot‐Mozo et al. 2024; Wettstein et al. 2019). Conversely, when the proportion of women in a study increased, the improvement in the MT group was 1.8 points higher than in the ET group for pain intensity, which could encourage further investigation into the difference in the type of therapeutic coping between women and men (Mercado et al. 2005; Ramond et al. 2011; Schmidt et al. 2001) as well as the role of gender stereotypes (Bizzoca et al. 2023; Prego‐Jimenez et al. 2022), influencing the evolution of CLBP (O'Sullivan et al. 2016; Samulowitz et al. 2018).

Sub‐analyses excluding Bronfort et al. (2011) and de Oliveira Meirelles et al. (2020) showed that MT provided a small short‐term benefit for pain intensity. In the analysis that included them, Bronfort et al. (2011), the study with the largest number of subjects showed inconclusive results. Therefore, when these studies were excluded, the short‐term statistical significance favoured MT, since Ulger et al. (2017), in favour of MT with a large size effect, and those articles showing a trend toward an effect acquire a higher weight. These findings suggest no robust conclusions, but they coincide with previous evidence (Coulter et al. 2018; Furlan et al. 2015; George et al. 2021), suggesting the benefit of joint mobilisations, manipulations and massage in short‐term pain management in CLBP. Some sources (Assendelft et al. 2004; Bronfort et al. 2008; Haas et al. 2004) have suggested that the effectiveness of the technique is not influenced by the type of therapist or the dosage of spinal manipulation, but rather by the expectation of improvement with MT (Bishop et al. 2011; Eklund et al. 2019). However, the relevance of this factor remains unclear (Donaldson et al. 2013).

The assessment of disability showed a statistically significant reduction with a small effect in favour of ET at the 6‐month follow‐up, which aligns with two previous reviews (Hayden, Ellis, Ogilvie, Malmivaara, et al. 2021; Saragiotto et al. 2016). Gomes‐Neto et al. reached the same conclusions, but as stated above, this synthesis considered studies with heterogeneous populations. In contrast, another review showed that long‐term follow‐up did not produce clinically significant changes (Saragiotto et al. 2016), and both reviews, in combination with our study, concluded that there were no statistically significant short‐ or medium‐term differences in the disability reduction. Meta‐regression showed that as the number of weeks of intervention increased, the improvement in the ET group was 0.42 points greater than that in the MT group for disability, which is consistent with the available literature (Krause et al. 2021; Metin Ökmen et al. 2017). Furthermore, when the proportion of females in the sample increased, the ET group improved by 3.93 points more than the MT group for disability, which is in line with the available literature on the benefit of ET over MT for the improvement of disability (Hayden, Ellis, Ogilvie, Malmivaara, et al. 2021; Saragiotto et al. 2016). However, it is important to note that patients with CLBP may require time to adapt and adhere to ET (Cecchi et al. 2012; Hicks et al. 2012; Nava‐Bringas et al. 2016), requiring a long follow‐up period to maintain stable clinical benefits (Gilanyi et al. 2024; Pinho et al. 2023; Shah et al. 2022). Excluding Bronfort et al. (2011) and de Oliveira Meirelles et al. (2020) did not significantly alter the results, which continued to show small long‐term differences in favour of ET for disability.

Furthermore, the analysis of physical function showed no statistically significant or clinically relevant differences between the ET and MT groups during the different measurements, consistent with previous findings (Saragiotto et al. 2016). Scientific evidence on the performance of ET and MT in the physical function outcomes for CLBP is limited, and previous studies have shown contradictory findings, with no statistically significant differences (Blanco‐Giménez et al. 2024) or claims of ET (Adorno and Brasil‐Neto 2013; Hidalgo et al. 2014) or MT (Cecchi et al. 2012) being superior in people with CLBP. Other studies (Jegan et al. 2017; Ünal et al. 2019) have shown that younger age predicts better marked improvements in physical function, which conditions the results and raises new questions about the influence of age on improvement in physical function. Sensitivity analysis showed that excluding Ulger et al. (2017) resulted in a small, yet statistically significant, improvement in physical function, favouring ET in the short term. This may be partly because, in the initial short‐term analysis, Ulger et al. (2017) described a more extensive manual intervention than in the rest of the study, which showed a large effect size favouring MT. Therefore, the exclusion of Ulger et al. (2017) displaced the previously existing short‐term effect favouring ET with Bronfort et al. (2011). This could have affected the direction of the effect and reduced between‐study heterogeneity, although one should be aware that the I^2^ statistic becomes less precise when analysing a small number of studies. These findings are consistent with previous literature (Hayden, Ellis, Ogilvie, Malmivaara, et al. 2021), which, like our secondary analysis, did not identify any differences compared with the MCID at the short term for ET in physical function. Accordingly, this is a good time to propose new lines of research that address the impact of ET and MT on the evolution of physical function in individuals with CLBP.

### 4.1 Clinical Implications

The results of this study may impact clinical practice by highlighting some individual characteristics that may influence the outcomes of people with CLBP receiving ET or MT. It will also allow methodological improvements in future RCTs on ET and MT prescription, proposing research on dosage parameters such as frequency, intensity, type, time, volume, progression and duration of both treatments or stratification into specific population subgroups (Vibe Fersum et al. 2013). In addition, the initial analysis of this study revealed that more research is required into the potential impact of biopsychosocial factors influenced by learning and culture (Samulowitz et al. 2018) on ET and MT outcomes. These factors could include gender stereotypes and roles and stratification into specific subgroups. Furthermore, other modifiable outcomes, such as the number of sessions, could help determine the optimal treatment approach. Clinically, our results support the notion that ET and MT share neurophysiological mechanisms that favour changes in symptomatology (Cook et al. 2024; McDevitt et al. 2023; Vigotsky and Bruhns 2015). Secondary analyses revealed the variety of existing ET and MT procedures, suggesting that excluding them from treatment may affect outcomes. Nevertheless, from the perspective of long‐term benefits, we should encourage research into treatments that allow us to improve our lifestyles, restore physical function and maintain them over time. It is important to show physicians that choosing between ET or MT is not necessarily effective. This can be achieved by prioritising multimodal treatment.

### 4.2 Limitations

In view of these results, some limitations must be highlighted. Despite the establishment of exclusion criteria and the potential inadequacy of the diagnostic criteria employed in the studies, the fundamental premise of these studies was based on the presence of pain. Therefore, we may have examined a heterogeneous population. Consequently, generalising our findings to the general population with CLBP must be approached cautiously. The number of RCTs performed in people with CLBP that isolated ET and MT is scarce, as is the sample size in some of them (Ferreira et al. 2010; de Oliveira Meirelles et al. 2020; Zhang et al. 2022). This, coupled with the exclusion of Bronfort et al. (2011) and de Oliveira Meirelles et al. (2020), may have hindered statistical power, decreased the reliability of the results and altered the precision of the I^2^ statistic in estimating heterogeneity. We aimed to raise awareness of the differences in the modalities of ET and MT approaches, as these variations may affect the results of the studies (Hidalgo et al. 2014). There is a lack of information regarding the dosage parameters of ET and MT. None of the studies reported the dosage of lumbopelvic motor control exercises. Only three studies reported general exercise dose (Bronfort et al. 2011; Ferreira et al. 2007, 2010). Only two studies (Ulger et al. 2017; Zhang et al. 2022) detailed the duration of the ET session, whereas another study (de Oliveira Meirelles et al. 2020) did not detail any of the characteristics of the TE programme. Regarding MT, only one study (Ulger et al. 2017) detailed the technique applied, constituting a problem in the clinical replication of the MT. The insufficient duration of treatment programmes and lack of adequate long‐term follow‐up may have also affected the quality of our results. In meta‐regression, the small number of observations resulted in an R^2^ value that did not accurately indicate the fit of the data, limiting the estimation of the disability models. In addition, the assessment of publication bias was not possible because of the lack of necessary studies included in the meta‐analyses (Ioannidis and Trikalinos 2007). Considering all facts, it is important to emphasise the need for future studies with improved inclusion criteria and prior validation of the underlying pain mechanisms.

### 4.3 Strengths

The strengths of our study are the use of systematic and replicable methods, which allowed us to robustly synthesise the available evidence and estimate the overall effect of the interventions more accurately. Furthermore, secondary analyses allowed us to isolate clinical reasoning approaches to MT, as well as propose future research on how different manual professions may influence clinical parameters in CLBP. Finally, meta‐regression allowed us to determine the influence of the initial predictors on the outcomes of the meta‐analysis. This revealed new research opportunities involving clinical trials that stratify specific population subgroups.

## 5 Conclusion

On the basis of our results, the current evidence is insufficient to definitively recommend ET over ET, or vice versa, for managing pain intensity, disability and physical function in people with CLBP. ET showed small long‐term benefits for disability in people with CLBP. MT showed small short‐term improvements in pain intensity when RCTs on the basis of osteopathic or chiropractic reasoning were excluded. There is a critical lack of high‐quality RCTs with rigorous methodological designs. Clinicians should consider the nuanced findings of this review when making treatment decisions for people with CLBP. Although both therapies alone might produce small beneficial effects on different clinical parameters, the choice between ET and MT should be reconsidered in favour of CPPP, considering individual patient factors, preferences, and the clinical context.

## Author Contributions

This study was designed by L.G.‐G., J.A.M.‐M. and Á.‐J.R.‐D. The experiments were performed by L.G.‐G., Á.‐J.R.‐D., A.C.‐M. and M.C.‐G. The data were analysed by L.G.‐G. and J.A.M.‐M., and the results were critically examined by all authors. L.G.‐G. had a primary role in preparing the manuscript, which was edited by J.A.M.‐M. and Á.‐J.R.‐D. All authors have approved the final version of the manuscript and agree to be accountable for all aspects of the work.

## Supporting information


**Data S1:** ejp70090‐sup‐0001‐Supinfo.pdf.

## References

[ejp70090-bib-0001] Adorno, M. L. G. R. , and J. P. Brasil‐Neto . 2013. “Assessment of the Quality of Life Through the SF‐36 Questionnaire in Patients With Chronic Nonspecific Low Back Pain.” Acta Ortopédica Brasileira 21: 202–207. 24453669 10.1590/S1413-78522013000400004 PMC3862010

[ejp70090-bib-0002] Ardern, C. L. , F. Büttner , R. Andrade , et al. 2022. “Implementing the 27 PRISMA 2020 Statement Items for Systematic Reviews in the Sport and Exercise Medicine, Musculoskeletal Rehabilitation and Sports Science Fields: The PERSiST (Implementing Prisma in Exercise, Rehabilitation, Sport Medicine and SporTs Science) Guidance.” British Journal of Sports Medicine 56: 175–195. 34625401 10.1136/bjsports-2021-103987 PMC8862073

[ejp70090-bib-0003] Assendelft, W. J. , S. C. Morton , E. I. Yu , M. J. Suttorp , and P. G. Shekelle . 2004. “Spinal Manipulative Therapy for Low‐Back Pain.” In Cochrane Database of Systematic Reviews, edited by W. J. Assendelft . John Wiley & Sons, Ltd. 10.1002/14651858.CD000447.pub2 14973958

[ejp70090-bib-0004] Balshem, H. , M. Helfand , H. J. Schünemann , et al. 2011. “GRADE Guidelines: 3. Rating the Quality of Evidence.” Journal of Clinical Epidemiology 64: 401–406. 21208779 10.1016/j.jclinepi.2010.07.015

[ejp70090-bib-0005] Bastiaens, F. , I. H. van de Wijgert , E. M. Bronkhorst , et al. 2024. “Factors Predicting Clinically Relevant Pain Relief After Spinal Cord Stimulation for Patients With Chronic Low Back and/or Leg Pain: A Systematic Review With Meta‐Analysis and Meta‐Regression.” Neuromodulation 27: 70–82. 38184342 10.1016/j.neurom.2023.10.188

[ejp70090-bib-0006] Bialosky, J. E. , J. M. Beneciuk , M. D. Bishop , et al. 2018. “Unraveling the Mechanisms of Manual Therapy: Modeling an Approach.” Journal of Orthopaedic and Sports Physical Therapy 48: 8–18. 29034802 10.2519/jospt.2018.7476

[ejp70090-bib-0007] Bishop, M. D. , J. E. Bialosky , and J. A. Cleland . 2011. “Patient Expectations of Benefit From Common Interventions for Low Back Pain and Effects on Outcome: Secondary Analysis of a Clinical Trial of Manual Therapy Interventions.” Journal of Manual and Manipulative Therapy 19: 20–25. 22294850 10.1179/106698110X12804993426929 PMC3172953

[ejp70090-bib-0008] Bizzoca, D. , G. Solarino , A. Pulcrano , et al. 2023. “Gender‐Related Issues in the Management of Low‐Back Pain: A Current Concepts Review.” Clinics and Practice 13: 1360–1368. 37987423 10.3390/clinpract13060122 PMC10660510

[ejp70090-bib-0009] Blanco‐Giménez, P. , J. Vicente‐Mampel , P. Gargallo , et al. 2024. “Clinical Relevance of Combined Treatment With Exercise in Patients With Chronic Low Back Pain: A Randomized Controlled Trial.” Scientific Reports 14: 17042. 39048701 10.1038/s41598-024-68192-2 PMC11269583

[ejp70090-bib-0010] Boekel, I. , A. L. Dutmer , H. R. Schiphorst Preuper , and M. F. Reneman . 2022. “Validation of the Work Ability Index‐Single Item and the Pain Disability Index‐Work Item in Patients With Chronic Low Back Pain.” European Spine Journal 31: 943–952. 35066684 10.1007/s00586-022-07109-x

[ejp70090-bib-0011] Bronfort, G. , M. Haas , R. Evans , G. Kawchuk , and S. Dagenais . 2008. “Evidence‐Informed Management of Chronic Low Back Pain With Spinal Manipulation and Mobilization.” Spine Journal 8: 213–225. 10.1016/j.spinee.2007.10.023 18164469

[ejp70090-bib-0012] Bronfort, G. , M. J. Maiers , R. L. Evans , et al. 2011. “Supervised Exercise, Spinal Manipulation, and Home Exercise for Chronic Low Back Pain: A Randomized Clinical Trial.” Spine Journal 11: 585–598. 10.1016/j.spinee.2011.01.036 21622028

[ejp70090-bib-0013] Cecchi, F. , S. Negrini , G. Pasquini , et al. 2012. “Predictors of Functional Outcome in Patients With Chronic Low Back Pain Undergoing Back School, Individual Physiotherapy or Spinal Manipulation.” European Journal of Physical and Rehabilitation Medicine 48: 371–378. 22569488

[ejp70090-bib-0014] Chiarotto, A. , L. J. Maxwell , R. W. Ostelo , M. Boers , P. Tugwell , and C. B. Terwee . 2019. “Measurement Properties of Visual Analogue Scale, Numeric Rating Scale, and Pain Severity Subscale of the Brief Pain Inventory in Patients With Low Back Pain: A Systematic Review.” Journal of Pain 20: 245–263. 30099210 10.1016/j.jpain.2018.07.009

[ejp70090-bib-0042] Cochrane Collaboration . 2020. Cochrane Handbook for Systematic Reviews of Interventions. Edited by J. P. T. Higgins , and J. Thomas . Wiley‐Blackwell.

[ejp70090-bib-0015] Cook, C. E. , D. Keter , W. T. Cade , B. A. Winkelstein , and W. R. Reed . 2024. “Manual Therapy and Exercise Effects on Inflammatory Cytokines: A Narrative Overview.” Frontiers in Rehabilitation Science 5: 1305925. 10.3389/fresc.2024.1305925 PMC11091266 38745971

[ejp70090-bib-0016] Corp, N. , G. Mansell , S. Stynes , et al. 2020. “Evidence‐Based Treatment Recommendations for Neck and Low Back Pain Across Europe: A Systematic Review of Guidelines.” European Journal of Pain 25: 275–295. 33064878 10.1002/ejp.1679 PMC7839780

[ejp70090-bib-0017] Coulter, I. D. , C. Crawford , E. L. Hurwitz , et al. 2018. “Manipulation and Mobilization for Treating Chronic Low Back Pain: A Systematic Review and Meta‐Analysis.” Spine Journal 18: 866–879. 10.1016/j.spinee.2018.01.013 PMC6020029 29371112

[ejp70090-bib-0018] de Oliveira Meirelles, F. , J. C. Oliveira Muniz Cunha , and E. B. da Silva . 2020. “Osteopathic Manipulation Treatment Versus Therapeutic Exercises in Patients With Chronic Nonspecific Low Back Pain: A Randomized, Controlled and Double‐Blind Study.” Journal of Back and Musculoskeletal Rehabilitation 33: 367–377. 31658037 10.3233/BMR-181355

[ejp70090-bib-0019] Donaldson, M. , K. Learman , B. O'Halloran , C. Showalter , and C. Cook . 2013. “The Role of Patients' Expectation of Appropriate Initial Manual Therapy Treatment in Outcomes for Patients With Low Back Pain.” Journal of Manipulative and Physiological Therapeutics 36: 276–283. 23829882 10.1016/j.jmpt.2013.05.016

[ejp70090-bib-0020] Draper‐Rodi, J. , T. Delion , A. MacMillan , et al. 2024. “Primary and Secondary Prevention of Musculoskeletal Pain and Disability in Chiropractic, Osteopathy, and Physiotherapy: A Scoping Review.” International Journal of Osteopathic Medicine 53: 100725.

[ejp70090-bib-0021] Dueñas, M. , J. A. Moral‐Munoz , J. Palomo‐Osuna , A. Salazar , H. De Sola , and I. Failde . 2020. “Differences in Physical and Psychological Health in Patients With Chronic Low Back Pain: A National Survey in General Spanish Population.” Quality of Life Research 29, no. 11: 2935–2947. 32556823 10.1007/s11136-020-02553-y

[ejp70090-bib-0022] Eklund, A. , D. De Carvalho , I. Pagé , et al. 2019. “Expectations Influence Treatment Outcomes in Patients With Low Back Pain. A Secondary Analysis of Data From a Randomized Clinical Trial.” European Journal of Pain 23: 1378–1389. 31034102 10.1002/ejp.1407 PMC6767754

[ejp70090-bib-0023] Etheridge, T. , G. P. Bostick , A. M. Hoens , et al. 2022. “Barriers to Physiotherapists' Use of Professional Development Tools for Chronic Pain: A Knowledge Translation Study.” Physiotherapy Canada 74: 355–362. 37324608 10.3138/ptc-2020-0148 PMC10262724

[ejp70090-bib-0024] Ferreira, M. , C. Maher , K. Refshauge , R. Herbert , and P. Hodges . 2010. “Changes in Recruitment of Transversus Abdominis Correlate With Disability in People With Chronic Low Back Pain.” British Journal of Sports Medicine 44: 1166–1172. 19474006 10.1136/bjsm.2009.061515

[ejp70090-bib-0025] Ferreira, M. L. 2009. “Relationship Between Spinal Stiffness and Outcome in Patients With Chronic Low Back Pain.” Manual Therapy 14, no. 1: 61–67. 18164644 10.1016/j.math.2007.09.013

[ejp70090-bib-0026] Ferreira, M. L. , P. H. Ferreira , J. Latimer , et al. 2007. “Comparison of General Exercise, Motor Control Exercise and Spinal Manipulative Therapy for Chronic Low Back Pain: A Randomized Trial.” Pain 131: 31–37. 17250965 10.1016/j.pain.2006.12.008

[ejp70090-bib-0027] Ferreira, M. L. , R. D. Herbert , P. H. Ferreira , et al. 2013. “The Smallest Worthwhile Effect of Nonsteroidal Anti‐Inflammatory Drugs and Physiotherapy for Chronic Low Back Pain: A Benefit‐Harm Trade‐Off Study.” Journal of Clinical Epidemiology 66: 1397–1404. 24021611 10.1016/j.jclinepi.2013.02.018

[ejp70090-bib-0028] Fiuza‐Luces, C. , N. Garatachea , N. A. Berger , and A. Lucia . 2013. “Exercise Is the Real Polypill.” Physiology (Bethesda) 28: 330–358. 23997192 10.1152/physiol.00019.2013

[ejp70090-bib-0029] Fullen, B. , B. Morlion , S. J. Linton , et al. 2022. “Management of Chronic Low Back Pain and the Impact on Patients' Personal and Professional Lives: Results From an International Patient Survey.” Pain Practice 22: 463–477. 35156770 10.1111/papr.13103 PMC9306505

[ejp70090-bib-0030] Furlan, A. D. , M. Giraldo , A. Baskwill , E. Irvin , and M. Imamura . 2015. “Massage for Low‐Back Pain.” Cochrane Database of Systematic Reviews 9: CD001929. 10.1002/14651858.CD001929.pub3 PMC8734598 26329399

[ejp70090-bib-0031] Gandek, B. , S. J. Sinclair , M. Kosinski , and J. E. Ware . 2004. “Psychometric Evaluation of the SF‐36 Health Survey in Medicare Managed Care.” Health Care Financing Review 25: 5. PMC4194895 15493441

[ejp70090-bib-0032] George, S. Z. , J. M. Fritz , S. P. Silfies , et al. 2021. “Interventions for the Management of Acute and Chronic Low Back Pain: Revision 2021.” Journal of Orthopaedic & Sports Physical Therapy 51: CPG1–CPG60. 10.2519/jospt.2021.0304 PMC10508241 34719942

[ejp70090-bib-0033] Gilanyi, Y. L. , B. Shah , A. G. Cashin , et al. 2024. “Barriers and Enablers to Exercise Adherence in People With Nonspecific Chronic Low Back Pain: A Systematic Review of Qualitative Evidence.” Pain 165: 2200–2214. 38635470 10.1097/j.pain.0000000000003234 PMC11404330

[ejp70090-bib-0034] Goldby, L. J. , A. P. Moore , J. Doust , and M. E. Trew . 2006. “A Randomized Controlled Trial Investigating the Efficiency of Musculoskeletal Physiotherapy on Chronic Low Back Disorder.” Spine 31: 1083–1093. 16648741 10.1097/01.brs.0000216464.37504.64

[ejp70090-bib-0035] Gomes‐Neto, M. , J. M. Lopes , C. S. Conceição , et al. 2017. “Stabilization Exercise Compared to General Exercises or Manual Therapy for the Management of Low Back Pain: A Systematic Review and Meta‐Analysis.” Physical Therapy in Sport 23: 136–142. 27707631 10.1016/j.ptsp.2016.08.004

[ejp70090-bib-0036] Gouteron, A. , A. Tabard‐Fougère , A. Bourredjem , J. M. Casillas , S. Armand , and S. Genevay . 2022. “The Flexion Relaxation Phenomenon in Nonspecific Chronic Low Back Pain: Prevalence, Reproducibility and Flexion‐Extension Ratios. A Systematic Review and Meta‐Analysis.” European Spine Journal 31: 136–151. 34553264 10.1007/s00586-021-06992-0

[ejp70090-bib-0037] Haas, M. , E. Groupp , M. Aickin , et al. 2004. “Dose Response for Chiropractic Care of Chronic Cervicogenic Headache and Associated Neck Pain: A Randomized Pilot Study.” Journal of Manipulative and Physiological Therapeutics 27: 547–553. 15614241 10.1016/j.jmpt.2004.10.007

[ejp70090-bib-0038] Hayden, J. A. , J. Ellis , R. Ogilvie , A. Malmivaara , and M. W. van Tulder . 2021. “Exercise Therapy for Chronic Low Back Pain.” Cochrane Database of Systematic Reviews 9: CD009790. 34580864 10.1002/14651858.CD009790.pub2 PMC8477273

[ejp70090-bib-0039] Hayden, J. A. , J. Ellis , R. Ogilvie , et al. 2021. “Some Types of Exercise Are More Effective Than Others in People With Chronic Low Back Pain: A Network Meta‐Analysis.” Journal of Physiotherapy 67: 252–262. 34538747 10.1016/j.jphys.2021.09.004

[ejp70090-bib-0040] Hicks, G. E. , F. Benvenuti , V. Fiaschi , et al. 2012. “Adherence to a Community‐Based Exercise Program Is a Strong Predictor of Improved Back Pain Status in Older Adults: An Observational Study.” Clinical Journal of Pain 28: 195–203. 21750458 10.1097/AJP.0b013e318226c411 PMC3274640

[ejp70090-bib-0041] Hidalgo, B. , C. Detrembleur , T. Hall , P. Mahaudens , and H. Nielens . 2014. “The Efficacy of Manual Therapy and Exercise for Different Stages of Non‐Specific Low Back Pain: An Update of Systematic Reviews.” Journal of Manual & Manipulative Therapy 22: 59–74. 24976749 10.1179/2042618613Y.0000000041 PMC4017797

[ejp70090-bib-0045] Ioannidis, J. P. A. , and T. A. Trikalinos . 2007. “The Appropriateness of Asymmetry Tests for Publication Bias in Meta‐Analyses: A Large Survey.” CMAJ 176: 1091–1096. 17420491 10.1503/cmaj.060410 PMC1839799

[ejp70090-bib-0053] Ipiña, S. L. , and A. I. Durand 2008. Inferencia estadistica y analisis de datos. Pearson Prentice Hall.

[ejp70090-bib-0046] Jegan, N. R. A. , M. Brugger , A. Viniol , et al. 2017. “Psychological Risk and Protective Factors for Disability in Chronic Low Back Pain ‐ a Longitudinal Analysis in Primary Care.” BMC Musculoskeletal Disorders 18: 114. 28320375 10.1186/s12891-017-1482-8 PMC5360090

[ejp70090-bib-0047] Jenks, A. , T. Hoekstra , M. van Tulder , R. W. Ostelo , S. M. Rubinstein , and A. Chiarotto . 2022. “Roland‐Morris Disability Questionnaire, Oswestry Disability Index, and Quebec Back Pain Disability Scale: Which has Superior Measurement Properties in Older Adults With Low Back Pain?” Journal of Orthopaedic and Sports Physical Therapy 52: 457–469. 35584027 10.2519/jospt.2022.10802

[ejp70090-bib-0048] Kaito, T. , Y. Matsuyama , T. Yamashita , et al. 2019. “Cost‐Effectiveness Analysis of the Pharmacological Management of Chronic Low Back Pain With Four Leading Drugs.” Journal of Orthopaedic Science 24: 805–811. 31230950 10.1016/j.jos.2019.06.004

[ejp70090-bib-0049] Kerry, R. , K. J. Young , D. W. Evans , et al. 2024. “A Modern Way to Teach and Practice Manual Therapy.” Chiropractic & Manual Therapies 32: 1–13. 38773515 10.1186/s12998-024-00537-0 PMC11110311

[ejp70090-bib-0050] Keter, D. L. , J. E. Bialosky , K. Brochetti , et al. 2025. “The Mechanisms of Manual Therapy: A Living Review of Systematic, Narrative, and Scoping Reviews.” PLoS One 20: e0319586. 40100908 10.1371/journal.pone.0319586 PMC11918397

[ejp70090-bib-0051] Krause, F. , D. Niederer , W. Banzer , and L. Vogt . 2021. “Medical Exercise and Physiotherapy Modes and Frequency as Predictors for a Recurrence of Chronic Non‐Specific Low Back Pain.” Journal of Back and Musculoskeletal Rehabilitation 34: 665–670. 33749637 10.3233/BMR-200149

[ejp70090-bib-0052] Kuithan, P. , N. R. Heneghan , A. Rushton , A. Sanderson , and D. Falla . 2019. “Lack of Exercise‐Induced Hypoalgesia to Repetitive Back Movement in People With Chronic Low Back Pain.” Pain Practice 19: 740–750. 31187932 10.1111/papr.12804

[ejp70090-bib-0054] Li, Y. , L. Yan , L. Hou , et al. 2023. “Exercise Intervention for Patients With Chronic Low Back Pain: A Systematic Review and Network Meta‐Analysis.” Frontiers in Public Health 11: 1155225. 38035307 10.3389/fpubh.2023.1155225 PMC10687566

[ejp70090-bib-0055] Manchikanti, L. , V. Singh , F. J. E. Falco , R. M. Benyamin , and J. A. Hirsch . 2014. “Epidemiology of Low Back Pain in Adults.” Neuromodulation 17, no. Suppl 2: 3–10. 25395111 10.1111/ner.12018

[ejp70090-bib-0056] McDevitt, A. W. , B. O'Halloran , and C. E. Cook . 2023. “Cracking the Code: Unveiling the Specific and Shared Mechanisms Behind Musculoskeletal Interventions.” Archives of Physiotherapy 13: 1–4. 37415258 10.1186/s40945-023-00168-3 PMC10327381

[ejp70090-bib-0057] Mercado, A. C. , L. J. Carroll , J. D. Cassidy , and P. Côté . 2005. “Passive Coping Is a Risk Factor for Disabling Neck or Low Back Pain.” Pain 117: 51–57. 16043291 10.1016/j.pain.2005.05.014

[ejp70090-bib-0058] Methley, A. M. , S. Campbell , C. Chew‐Graham , R. McNally , and S. Cheraghi‐Sohi . 2014. “PICO, PICOS and SPIDER: A Comparison Study of Specificity and Sensitivity in Three Search Tools for Qualitative Systematic Reviews.” BMC Health Services Research 14: 579. 25413154 10.1186/s12913-014-0579-0 PMC4310146

[ejp70090-bib-0059] Metin Ökmen, B. , E. Koyuncu , B. Uysal , and N. Özgirgİn . 2017. “The Effects of the Number of Physical Therapy Sessions on Pain, Disability,and Quality of Life in Patients With Chronic Low Back Pain.” Turkish Journal of Medical Sciences 47: 1425–1431. 29151313 10.3906/sag-1607-78

[ejp70090-bib-0060] Meucci, R. D. , A. G. Fassa , and N. M. Xavier Faria . 2015. “Prevalence of Chronic Low Back Pain: Systematic Review.” Revista de Saúde Pública 49: 73. 10.1590/S0034-8910.2015049005874 PMC4603263 26487293

[ejp70090-bib-0061] Moniz, A. , S. T. Duarte , P. Aguiar , et al. 2024. “Physiotherapists' Barriers and Facilitators to the Implementation of a Behaviour Change‐Informed Exercise Intervention to Promote the Adoption of Regular Exercise Practice in Patients at Risk of Recurrence of Low Back Pain: A Qualitative Study.” BMC Primary Care 25: 39. 38279123 10.1186/s12875-024-02274-y PMC10811813

[ejp70090-bib-0062] Moreno‐Ligero, M. , J. A. Moral‐Munoz , I. Failde , and M. Dueñas . 2023. “Physical Activity Levels in Adults With Chronic Low Back Pain: A National Survey in the General Spanish Population.” Journal of Rehabilitation Medicine 55: jrm00366. 36661849 10.2340/jrm.v55.4352 PMC9881013

[ejp70090-bib-0063] Murad, M. H. , Z. Wang , H. Chu , and L. Lin . 2019. “When Continuous Outcomes Are Measured Using Different Scales: Guide for Meta‐Analysis and Interpretation.” BMJ (Clinical Research Ed.) 364: k4817. 10.1136/bmj.k4817 PMC6890471 30670455

[ejp70090-bib-0064] Nava‐Bringas, T. I. , A. Roeniger‐Desatnik , A. Arellano‐Hernández , and E. Cruz‐Medina . 2016. “Adherence to a Stability Exercise Program in Patients With Chronic Low Back Pain.” Cirugia y Cirujanos 84: 384–391. 26769530 10.1016/j.circir.2015.10.014

[ejp70090-bib-0065] NICE . 2017. NICE Endorsed Resource—National Pathway of Care for Low Back and Radicular Pain|Low Back Pain and Sciatica in Over 16s: Assessment and Management|Guidance. NICE.

[ejp70090-bib-0066] NICE . 2020. NICE Overview|Low Back Pain and Sciatica in Over 16s: Assessment and Management|Guidance. NICE. 33090750

[ejp70090-bib-0067] Nugent, S. M. , T. I. Lovejoy , S. Shull , S. K. Dobscha , and B. J. Morasco . 2021. “Associations of Pain Numeric Rating Scale Scores Collected During Usual Care With Research Administered Patient Reported Pain Outcomes.” Pain Medicine 22: 2235–2241. 33749760 10.1093/pm/pnab110 PMC8677438

[ejp70090-bib-0068] O'Sullivan, P. , J. P. Caneiro , M. O'Keeffe , and K. O'Sullivan . 2016. “Unraveling the Complexity of Low Back Pain.” Journal of Orthopaedic and Sports Physical Therapy 46: 932–937. 27802794 10.2519/jospt.2016.0609

[ejp70090-bib-0069] Owen, P. J. , C. T. Miller , N. L. Mundell , et al. 2020. “Which Specific Modes of Exercise Training Are Most Effective for Treating Low Back Pain? Network Meta‐Analysis.” British Journal of Sports Medicine 54: 1279–1287. 31666220 10.1136/bjsports-2019-100886 PMC7588406

[ejp70090-bib-0070] Pericot‐Mozo, X. , R. Suñer‐Soler , G. Reig‐Garcia , et al. 2024. “Quality of Life in Patients With Chronic Low Back Pain and Differences by Sex: A Longitudinal Study.” Journal of Personalized Medicine 14: 496. 38793078 10.3390/jpm14050496 PMC11121820

[ejp70090-bib-0071] Pinho, H. , M. Neves , F. Costa , and A. G. Silva . 2023. “Pain Intensity and Pain Sensitivity Are Not Increased by a Single Session of High‐Intensity Interval Aerobic Exercise in Individuals With Chronic Low Back Pain: A Randomized and Controlled Trial.” Musculoskeletal Science & Practice 66: 102824. 37421759 10.1016/j.msksp.2023.102824

[ejp70090-bib-0072] Prego‐Jimenez, S. , E. Pereda‐Pereda , J. Perez‐Tejada , J. Aliri , O. Goñi‐Balentziaga , and A. Labaka . 2022. “The Impact of Sexism and Gender Stereotypes on the Legitimization of Women's Low Back Pain.” Pain Management Nursing 23: 591–595. 35428592 10.1016/j.pmn.2022.03.008

[ejp70090-bib-0073] Qaseem, A. , F. Ma , D. Td , et al. 2017. “Noninvasive Treatments for Acute, Subacute, and Chronic Low Back Pain: A Clinical Practice Guideline From the American College of Physicians.” Annals of Internal Medicine 166: 514–530. 28192789 10.7326/M16-2367

[ejp70090-bib-0074] Ramond, A. , C. Bouton , I. Richard , et al. 2011. “Psychosocial Risk Factors for Chronic Low Back Pain in Primary Care—A Systematic Review.” Family Practice 28: 12–21. 20833704 10.1093/fampra/cmq072

[ejp70090-bib-0075] Rasmussen‐Barr, E. , L. Nilsson‐Wikmar , and I. Arvidsson . 2003. “Stabilizing Training Compared With Manual Treatment in Sub‐Acute and Chronic Low‐Back Pain.” Manual Therapy 8: 233–241. 14559046 10.1016/s1356-689x(03)00053-5

[ejp70090-bib-0076] Rice, D. , J. Nijs , E. Kosek , et al. 2019. “Exercise‐Induced Hypoalgesia in Pain‐Free and Chronic Pain Populations: State of the Art and Future Directions.” Journal of Pain 20: 1249–1266. 30904519 10.1016/j.jpain.2019.03.005

[ejp70090-bib-0077] Samulowitz, A. , I. Gremyr , E. Eriksson , and G. Hensing . 2018. ““Brave Men” and “Emotional Women”: A Theory‐Guided Literature Review on Gender Bias in Health Care and Gendered Norms Towards Patients With Chronic Pain.” Pain Research & Management 2018: 6358624. 29682130 10.1155/2018/6358624 PMC5845507

[ejp70090-bib-0078] Saragiotto, B. T. , C. G. Maher , T. P. Yamato , et al. 2016. “Motor Control Exercise for Chronic Non‐Specific Low‐Back Pain.” Cochrane Database of Systematic Reviews 2016: CD012004. 26742533 10.1002/14651858.CD012004 PMC8761501

[ejp70090-bib-0079] Schmidt, B. , P. Kolip , and B. Greitemann . 2001. “Gender‐Specific Aspects in Chronic Low Back Pain Rehabilitation.” Rehabilitation (Stuttg) 40: 261–266. 11579372 10.1055/s-2001-17414

[ejp70090-bib-0080] Shah, B. , M. A. Wewege , Y. L. Gilanyi , et al. 2022. “Effects of a Single Exercise Session on Pain Intensity in Adults With Chronic Pain: A Systematic Review and Meta‐Analysis.” Musculoskeletal Science & Practice 62: 102679. 36332334 10.1016/j.msksp.2022.102679

[ejp70090-bib-0081] Shivachev, Y. , and P. Mancheva . 2022. “Manual Therapy and Osteopathy ‐ Comparative Analysis.” Journal of IMAB—Annual Proceeding Scientific Papers 28: 4233–4236.

[ejp70090-bib-0082] Silvernail, J. L. , G. D. Deyle , G. M. Jensen , et al. 2024. “Orthopaedic Manual Physical Therapy: A Modern Definition and Description.” Physical Therapy 104: pzae036. 38457654 10.1093/ptj/pzae036

[ejp70090-bib-0083] Soriano, J. B. , D. Rojas‐Rueda , J. Alonso , et al. 2018. “The Burden of Disease in Spain: Results From the Global Burden of Disease 2016.” Medicina Clínica 151: 171–190. 30037695 10.1016/j.medcli.2018.05.011

[ejp70090-bib-0084] Tomschi, F. , A. Zschunke , and T. Hilberg . 2025. “Ten Minutes of Core Stabilisation Exercise Result in Local Exercise‐Induced Hypoalgesia in Patients With Chronic Unspecific Low Back Pain.” European Journal of Pain 29: e4794. 39923121 10.1002/ejp.4794 PMC11807238

[ejp70090-bib-0085] Ulger, O. , A. Demirel , M. Oz , and S. Tamer . 2017. “The Effect of Manual Therapy and Exercise in Patients With Chronic Low Back Pain: Double Blind Randomized Controlled Trial.” Journal of Back and Musculoskeletal Rehabilitation 30: 1303–1309. 28946522 10.3233/BMR-169673

[ejp70090-bib-0086] Ünal, Ö. , Y. Akyol , B. Tander , Y. Ulus , Y. Terzi , and Ö. Kuru . 2019. “The Relationship of Illness Perceptions With Demographic Features, Pain Severity, Functional Capacity, Disability, Depression, and Quality of Life in Patients With Chronic Low Back Pain.” Turkish Journal of Physical Medicine and Rehabilitation 65: 301–308. 31893266 10.5606/tftrd.2019.3248 PMC6935732

[ejp70090-bib-0087] Vaegter, H. B. , and M. D. Jones . 2020. “Exercise‐Induced Hypoalgesia After Acute and Regular Exercise: Experimental and Clinical Manifestations and Possible Mechanisms in Individuals With and Without Pain.” Pain Reports 5: E823. 33062901 10.1097/PR9.0000000000000823 PMC7523781

[ejp70090-bib-0088] Vibe Fersum, K. , P. O'Sullivan , J. S. Skouen , A. Smith , and A. Kvåle . 2013. “Efficacy of Classification‐Based Cognitive Functional Therapy in Patients With Non‐Specific Chronic Low Back Pain: A Randomized Controlled Trial.” European Journal of Pain 17: 916–928. 23208945 10.1002/j.1532-2149.2012.00252.x PMC3796866

[ejp70090-bib-0089] Vigotsky, A. D. , and R. P. Bruhns . 2015. “The Role of Descending Modulation in Manual Therapy and Its Analgesic Implications: A Narrative Review.” Pain Research and Treatment 2015: 292805. 26788367 10.1155/2015/292805 PMC4695672

[ejp70090-bib-0090] Wayne, P. M. , J. E. Buring , D. M. Eisenberg , et al. 2019. “Cost‐Effectiveness of a Team‐Based Integrative Medicine Approach to the Treatment of Back Pain.” Journal of Alternative and Complementary Medicine 25: S138–S146. 30870015 10.1089/acm.2018.0503 PMC6444892

[ejp70090-bib-0091] Wettstein, M. , W. Eich , C. Bieber , and J. Tesarz . 2019. “Pain Intensity, Disability, and Quality of Life in Patients With Chronic Low Back Pain: Does Age Matter?” Pain Medicine 20: 464–475. 29701812 10.1093/pm/pny062 PMC6387985

[ejp70090-bib-0092] Wewege, M. A. , and M. D. Jones . 2021. “Exercise‐Induced Hypoalgesia in Healthy Individuals and People With Chronic Musculoskeletal Pain: A Systematic Review and Meta‐Analysis.” Journal of Pain 22: 21–31. 32599154 10.1016/j.jpain.2020.04.003

[ejp70090-bib-0093] Zhang, Z. , C. Zhang , Y. Li , C. Wang , and Q. Yu . 2022. “Lipid and Metabolic Alteration Involvement in Physiotherapy for Chronic Nonspecific Low Back Pain.” Lipids in Health and Disease 21: 125. 36434687 10.1186/s12944-022-01737-4 PMC9700977

