# Supplementary material for: Exercise Therapy Versus Manual Therapy for the Management of Pain Intensity, Disability, and Physical Function in People With Chronic Low Back Pain: A Systematic Review With Meta‐Analysis and Meta‐Regression
Source: Eur J Pain. 2025 Aug 1;29(8):e70090. doi: 10.1002/ejp.70090 (PMC12314856; doi:10.1002/ejp.70090)
Supplement: Supplementary file 1 — Data S1: ejp70090‐sup‐0001‐Supinfo.pdf. [file EJP-29-0-s001.pdf]

Exercise therapy versus manual therapy for the management of pain intensity, disability, and physical function in people with chronic low back pain: a systematic review with meta-analysis and meta-regression

Supplemental Materials	Page
**Figure S1**. Risk of bias assessment: risk of bias summary (A).	2
**Figure S2**. Risk of bias assessment: risk of bias graph (B).	2
**Figure S3-7**. Forest plots for each comparison.	3
**Table S1.** PICOS criteria, MeSH terms, and search strings.	6
**Table S2.** Evidence synthesis of outcomes based on certainty and importance using the GRADE method.	10
**Table S3-S10**. Sensitivity analysis to assess the impact of methodology and characteristics of included studies.	10

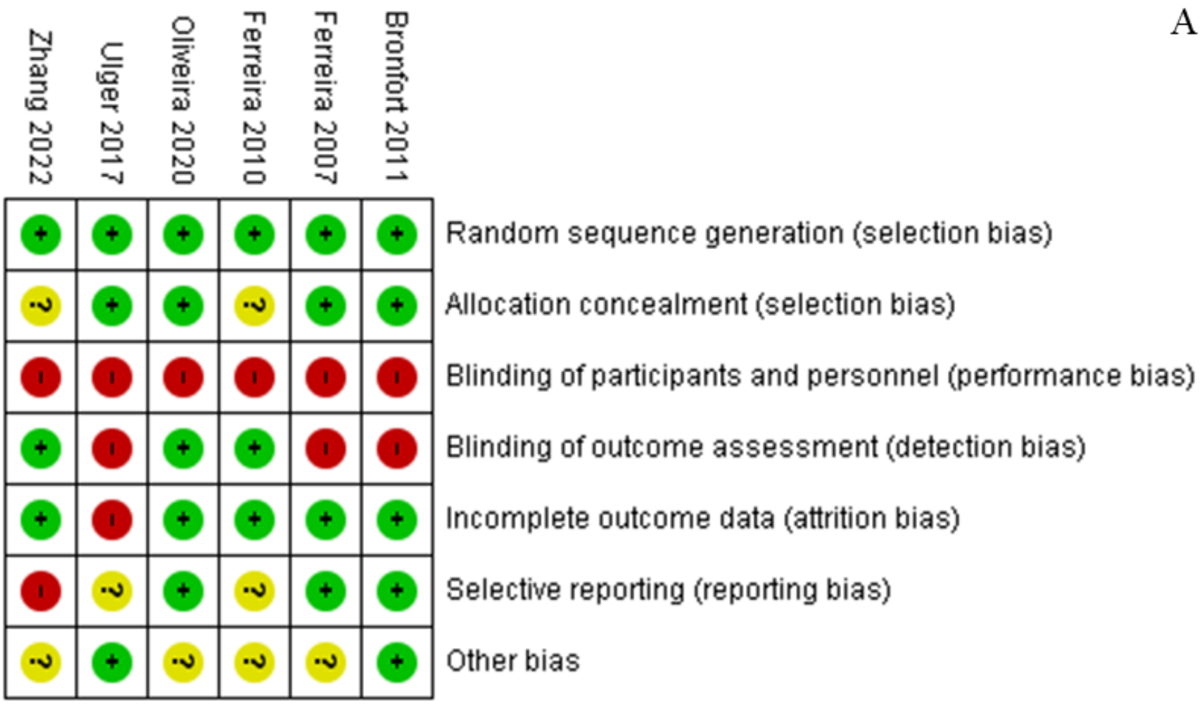

Figure S1. Risk of bias assessment: risk of bias summary (A).

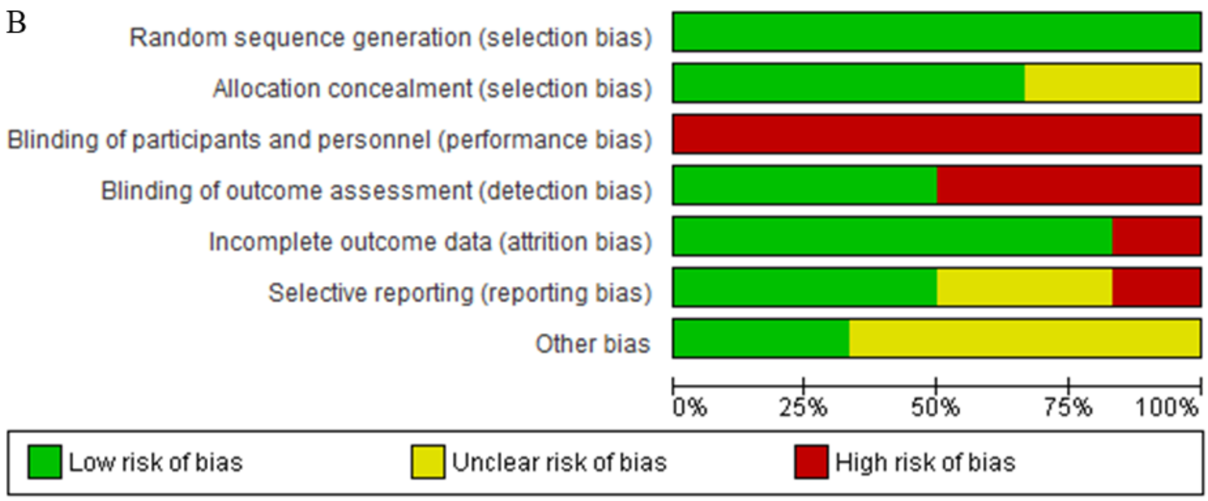

Figure S2. Risk of bias assessment: risk of bias graph (B).

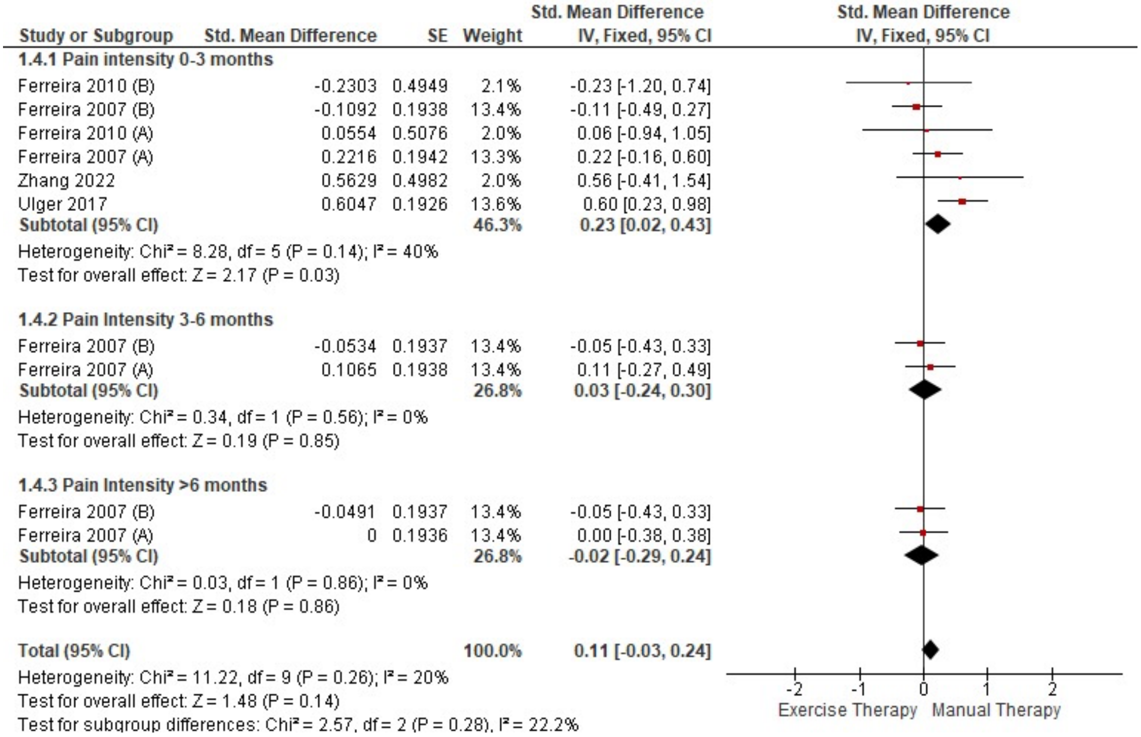

Figure S3. Forest plot of the meta-analysis for pain intensity, excluding Oliveira et al. and Bronfort et al.

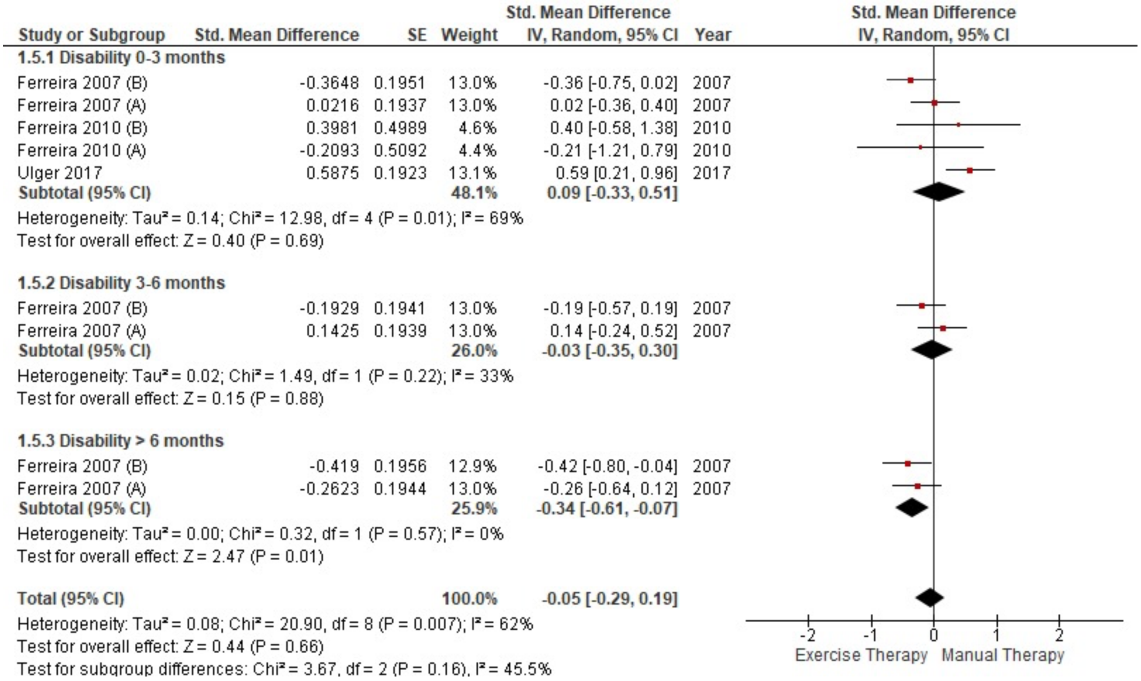

Figure S4. Forest plot of the meta-analysis of disability, excluding Oliveira et al. and Bronfort et al.

Study or Subgroup	Exercise Therapy Mean	SD	Total	Manual Therapy Mean	SD	Total	Weight	Mean Difference IV, Random, 95% CI
**1.1.1 Pain Intensity 0-3 months**								
Bronfort 2011 (A)	-1.4	1.28	100	-1.5	1.3	50	8.0%	0.10 [-0.34, 0.54]
Bronfort 2011 (B)	-1.6	1.43	101	-1.5	1.3	50	7.9%	-0.10 [-0.56, 0.36]
Ferreira 2007 (A)	-1.7	1.76	80	-2.1	1.86	40	6.5%	0.40 [-0.29, 1.09]
Ferreira 2007 (B)	-2.3	1.8	80	-2.1	1.86	40	6.4%	-0.20 [-0.90, 0.50]
Ferreira 2010 (A)	-2.36	1.77	11	-2.46	1.59	7	2.8%	0.10 [-1.48, 1.68]
Ferreira 2010 (B)	-2.8	1.26	10	-2.46	1.59	6	3.0%	-0.34 [-1.83, 1.15]
Oliveira 2020	-1.8	1.57	18	-4.9	1.31	20	5.2%	3.10 [2.17, 4.03]
Ulger 2017	-2.27	1.93	56	-3.27	1.3	57	7.0%	1.00 [0.39, 1.61]
Zhang 2022	-2.16	1.43	9	-2.96	1.25	8	3.7%	0.80 [-0.47, 2.07]
**Subtotal (95% CI)**			**465**			**278**	**50.4%**	**0.55 [-0.06, 1.15]**
Heterogeneity: Tau² = 0.64; Chi² = 47.11, df = 8 (P < 0.00001); I² = 83%								
Test for overall effect: Z = 1.78 (P = 0.08)								
**1.1.2 Pain Intensity 3-6 months**								
Bronfort 2011 (A)	2.5	1.5	100	2.5	1.36	100	8.3%	0.00 [-0.40, 0.40]
Bronfort 2011 (B)	-2	1.57	101	-2.5	1.36	100	8.2%	0.50 [0.09, 0.91]
Ferreira 2007 (A)	-1.7	1.87	80	-1.9	1.86	40	6.4%	0.20 [-0.51, 0.91]
Ferreira 2007 (B)	-2	1.86	80	-1.9	1.86	40	6.4%	-0.10 [-0.81, 0.61]
**Subtotal (95% CI)**			**361**			**280**	**29.3%**	**0.19 [-0.10, 0.48]**
Heterogeneity: Tau² = 0.02; Chi² = 3.77, df = 3 (P = 0.29); I² = 20%								
Test for overall effect: Z = 1.30 (P = 0.19)								
**1.1.3 Pain Intensity > 6 months**								
Bronfort 2011 (A)	-2.2	1.5	100	-2.1	1.72	100	8.0%	-0.10 [-0.55, 0.35]
Bronfort 2011 (B)	2.1	1.5	101	2.1	1.72	100		Not estimable
Ferreira 2007 (A)	1.3	2	80	1.3	1.93	40	6.2%	0.00 [-0.74, 0.74]
Ferreira 2007 (B)	-1.4	2.07	80	-1.3	1.93	40	6.1%	-0.10 [-0.85, 0.65]
**Subtotal (95% CI)**			**260**			**180**	**20.3%**	**-0.08 [-0.42, 0.26]**
Heterogeneity: Tau² = 0.00; Chi² = 0.06, df = 2 (P = 0.97); I² = 0%								
Test for overall effect: Z = 0.45 (P = 0.65)								
**Total (95% CI)**			**1086**			**738**	**100.0%**	**0.30 [-0.01, 0.61]**
Heterogeneity: Tau² = 0.26; Chi² = 55.55, df = 15 (P < 0.00001); I² = 73%								
Test for overall effect: Z = 1.89 (P = 0.06)								
Test for subgroup differences: Chi² = 3.44, df = 2 (P = 0.18), I² = 41.9%								

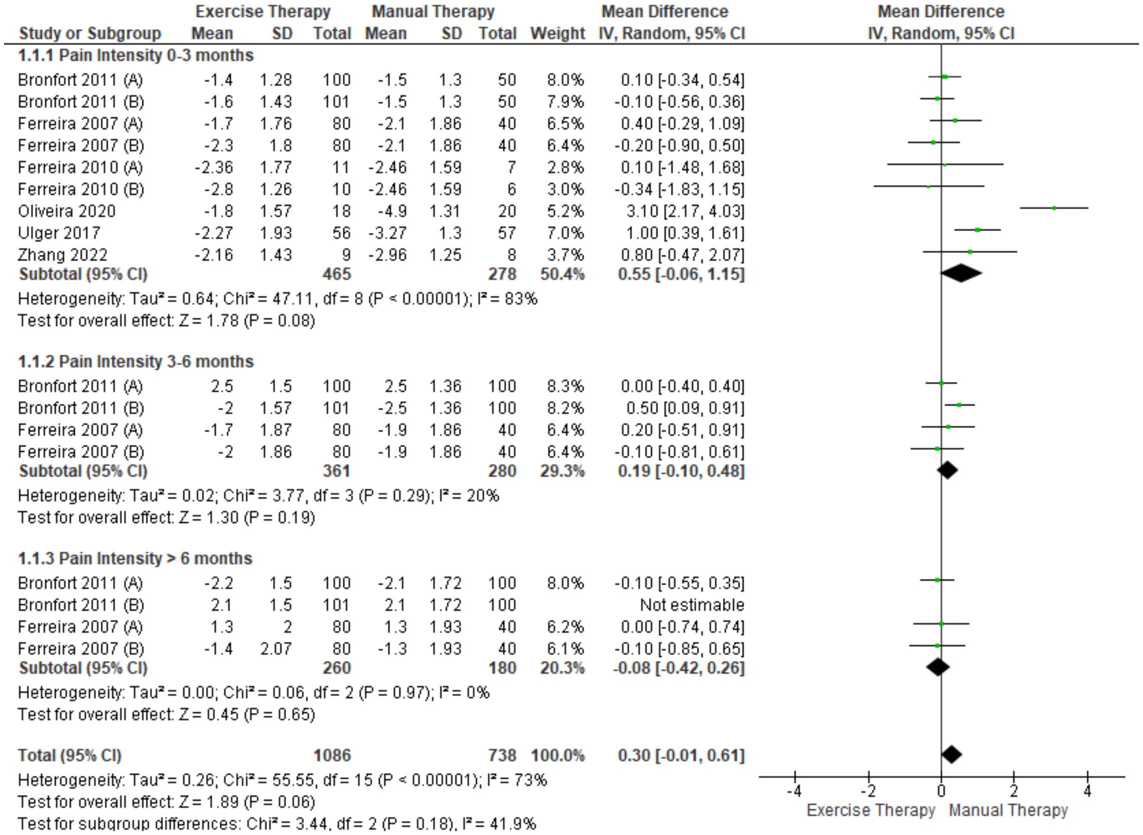

Figure S5. Forest plot of the meta-analysis for MCID in pain intensity.

Study or Subgroup	Exercise Therapy Mean	SD	Total	Manual Therapy Mean	SD	Total	Weight	Mean Difference IV, Fixed, 95% CI
**1.1.1 Pain Intensity 0-3 months**								
Ferreira 2007 (A)	-1.7	1.76	80	-2.1	1.86	40	13.1%	0.40 [-0.29, 1.09]
Ferreira 2007 (B)	-2.3	1.8	80	-2.1	1.86	40	12.9%	-0.20 [-0.90, 0.50]
Ferreira 2010 (A)	-2.36	1.77	11	-2.46	1.59	7	2.5%	0.10 [-1.48, 1.68]
Ferreira 2010 (B)	-2.8	1.26	10	-2.46	1.59	6	2.8%	-0.34 [-1.83, 1.15]
Ulger 2017	-2.27	1.93	56	-3.27	1.3	57	17.0%	1.00 [0.39, 1.61]
Zhang 2022	-2.16	1.43	9	-2.96	1.25	8	3.9%	0.80 [-0.47, 2.07]
**Subtotal (95% CI)**			**246**			**158**	**52.2%**	**0.42 [0.08, 0.77]**
Heterogeneity: Chi² = 8.02, df = 5 (P = 0.15); I² = 38%								
Test for overall effect: Z = 2.39 (P = 0.02)								
**1.1.2 Pain Intensity 3-6 months**								
Ferreira 2007 (A)	-1.7	1.87	80	-1.9	1.86	40	12.6%	0.20 [-0.51, 0.91]
Ferreira 2007 (B)	-2	1.86	80	-1.9	1.86	40	12.6%	-0.10 [-0.81, 0.61]
**Subtotal (95% CI)**			**160**			**80**	**25.2%**	**0.05 [-0.45, 0.55]**
Heterogeneity: Chi² = 0.35, df = 1 (P = 0.56); I² = 0%								
Test for overall effect: Z = 0.20 (P = 0.85)								
**1.1.3 Pain Intensity > 6 months**								
Ferreira 2007 (A)	1.3	2	80	1.3	1.93	40	11.4%	0.00 [-0.74, 0.74]
Ferreira 2007 (B)	-1.4	2.07	80	-1.3	1.93	40	11.2%	-0.10 [-0.85, 0.65]
**Subtotal (95% CI)**			**160**			**80**	**22.6%**	**-0.05 [-0.58, 0.48]**
Heterogeneity: Chi² = 0.03, df = 1 (P = 0.85); I² = 0%								
Test for overall effect: Z = 0.18 (P = 0.85)								
**Total (95% CI)**			**566**			**318**	**100.0%**	**0.22 [-0.03, 0.47]**
Heterogeneity: Chi² = 11.16, df = 9 (P = 0.26); I² = 19%								
Test for overall effect: Z = 1.74 (P = 0.08)								
Test for subgroup differences: Chi² = 2.76, df = 2 (P = 0.25), I² = 27.5%								

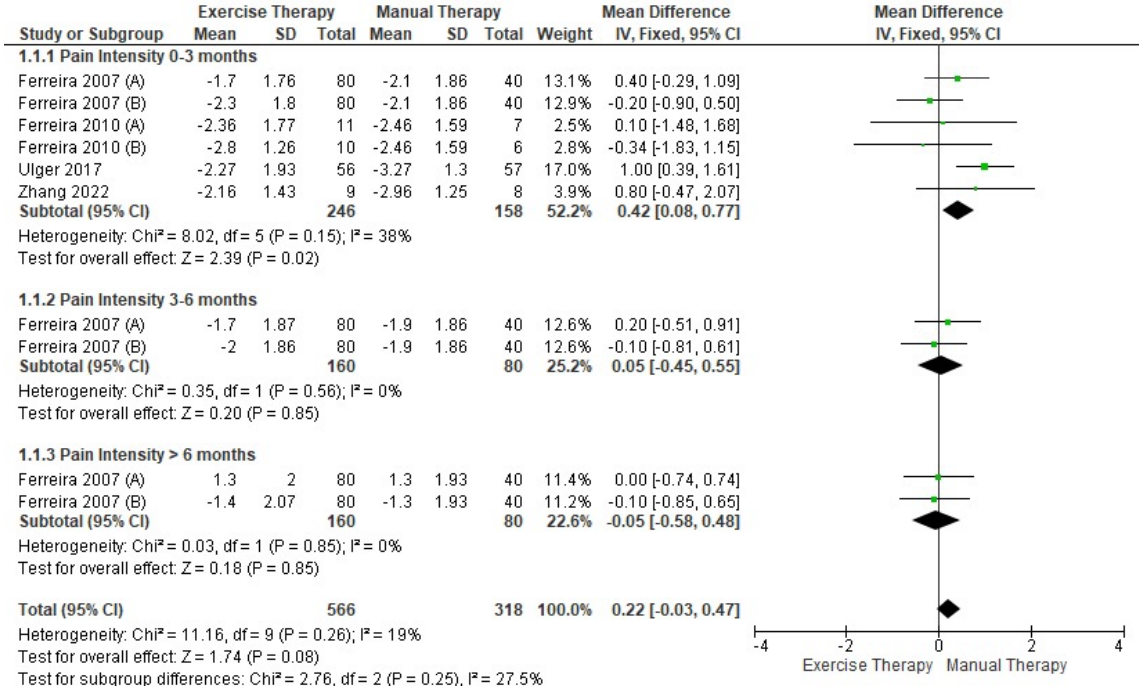

Figure S6. Forest plot of the meta-analysis of MCID for pain intensity, excluding Oliveira et al. and Bronfort et al.

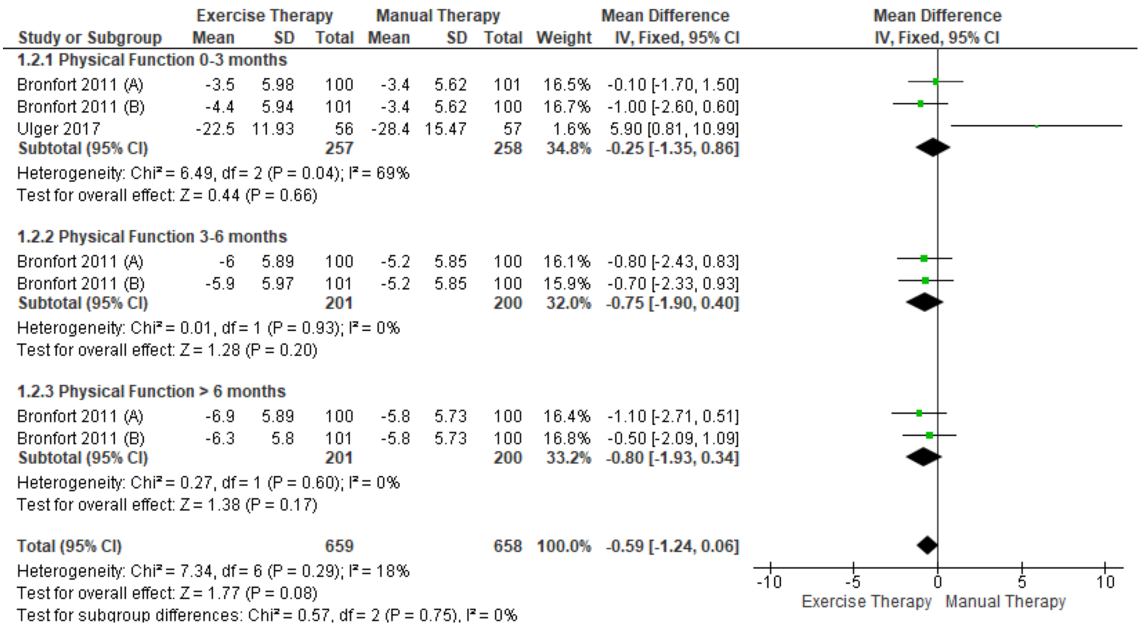

Figure S7. Forest plot of the meta-analysis for MCID in physical function.

Table S1. PICOS criteria, MeSH terms, and search strings.

**PICOS CRITERIA**<br>P: "Low Back Pain"[Mesh]<br>I: "Exercise Therapy"[Mesh]<br>C: "Musculoskeletal Manipulations"[Mesh]<br>O: "Musculoskeletal Pain"[Mesh] // "Quality of Life"[Mesh] // "Disabled Persons"[Mesh], "Disability"[Tiab]<br>S: "Randomized Controlled Trial"[Mesh]
**MeSH TERMS**<br>**P:** "Low Back Pain"[Mesh] OR "Chronic Low Back Pain" OR "Nociplastic Low Back Pain" OR "Non-specific Low Back Pain"<br>**I:** "Exercise Therapy"[Mesh] OR (Exercise Therapy[MeSH]) OR (Exercise Therap*) OR (Rehabilitation Exercise*)<br>Strength training: Resistance Training[MeSH] OR Strength Training[Tiab] OR Weight-Lifting Exercise* Program*[Tiab]<br>Resistance: Endurance Training[MeSH]<br>Aquatic therapy: Aquatic Therap* OR Pool Therap*[Tiab]<br>Motor Control: (Sensorimotor Exercise[TIAB]) OR (Sensorimotor Training[TIAB])<br>CORE: (Core Balance[TIAB]) OR (Core Stability[TIAB])<br>Pilates: (Exercise* Movement* Technique*[TIAB]) OR (Pilates Training[TIAB])<br>Yoga: (Yoga[TIAB])<br>Tai-Chi: (Tai Ji[TIAB]) OR (Tai Chi[TIAB])<br>Stretching: (Muscle Stretching Exercise*[TIAB]) OR (Proprioceptive Neuromuscular Facilitation[TIAB]) OR (PNF Stretching[TIAB])<br>Breathing: (Breathing Exercise*[TIAB]) OR (Respiratory Muscle Training[TIAB])<br>**C:** "Musculoskeletal Manipulations"[Mesh]<br>Manual therapy: (Musculoskeletal Manipulations[MH]) OR (Musculoskeletal Manipulation*[TIAB]) OR (Craniosacral Massage[TIAB]) OR (Reflexology[TIAB]) OR (Manipulation Therap*[TIAB]) OR (Manual Therap*[TIAB])<br>Osteopathy: (Manipulation, Osteopathic[MH] OR (Manipulation, Osteopathic[TIAB]) OR (Osteopathic Manipulative Treatment*[TIAB])<br>Chiropractic: (Chiropractic Manipulation[TIAB]) OR (Chiropractic Spinal Adjustment*[TIAB])

Neural mobilization: (Neurodynamic Mobilization*[TIAB]) OR (Neurodynamic Technique*[TIAB])

Mulligan Technique: (Mulligan Mobilization[TIAB]) OR (Mobilization With Movement[TIAB])

Cyriax Method: (Cyriax Friction Massage[TIAB])

Myofascial therapy: (Myofascial Release Therap*[TIAB])

Maitland Method: (Maitland Mobilization[TIAB])

O: "Musculoskeletal Pain"[Mesh] // "Quality of Life"[Mesh] // "Disabled Persons"[Mesh], "Disability"[Tiab]

S: "Randomized Controlled Trial"[Mesh] OR "Clinical Trial" OR "Controlled Trial" OR "Randomized Controlled Trial"

SEARCH STRINGS

Database	Descriptors	Results	Final articles
Medline	((Low Back Pain[MH]) OR (Low Back Pain[TIAB]) OR (Chronic Low Back Pain[TIAB]) OR (Nociplastic Low Back Pain[TIAB]) OR (Nociplastic Pain[TIAB]) OR (Non-specific Low Back Pain[TIAB])) AND ((Exercise Therapy[MH]) OR (Exercise Therap*[TIAB]) OR (Rehabilitation Exercise*[TIAB]) OR (Resistance Training[TIAB]) OR (Strength Training[TIAB]) OR (Weight-Lifting Exercise Program*[TIAB]) OR (Endurance Training[TIAB]) OR (Aquatic Therap*[TIAB]) OR (Pool Therap*[TIAB]) OR (Sensorimotor Exercise[TIAB]) OR (Sensorimotor Training[TIAB]) OR (Core Balance[TIAB]) OR (Core Stability[TIAB]) OR (Exercise Movement Technique*[TIAB]) OR (Pilates Training[TIAB]) OR (Yoga[TIAB]) OR (Tai Ji[TIAB]) OR (Tai Chi[TIAB]) OR (Muscle Stretching Exercise*[TIAB]) OR (Proprioceptive Neuromuscular Facilitation[TIAB]) OR (PNF Stretching[TIAB]) OR (Breathing Exercise*[TIAB]) OR (Respiratory Muscle Training[TIAB]) OR (McKenzie Method[TIAB])) AND ((Musculoskeletal Manipulations[MH]) OR (Musculoskeletal Manipulation*[TIAB]) OR (Craniosacral Massage[TIAB]) OR (Reflexology[TIAB]) OR (Manipulation Therap*[TIAB]) OR (Manual Therap*[TIAB]) OR (Manipulation, Osteopathic[MH] OR (Manipulation, Osteopathic[TIAB]) OR (Osteopathic Manipulative Treatment*[TIAB]) OR (Chiropractic Manipulation[TIAB]) OR (Chiropractic Spinal Adjustment*[TIAB]) OR (Neurodynamic Mobilization*[TIAB]) OR (Neurodynamic Technique*[TIAB]) OR (Mulligan Mobilization[TIAB]) OR (Mobilization With	513	1

	Movement[TIAB]) OR (Cyriax Friction Massage[TIAB]) OR (Myofascial Release Therap*[TIAB]) OR (Maitland Mobilization[TIAB])) AND ((Musculoskeletal Pain[MH]) OR (Quality of Life[MH]) OR (Disability[Tiab]) OR (Sleep Quality[MH])) AND ((Clinical Trial[TIAB]) OR (Controlled Trial[TIAB]) OR (Randomized Controlled Trial[TIAB]))		
Cochrane Library	((Low Back Pain[MH]) OR (Low Back Pain[TIAB]) OR (Chronic Low Back Pain[TIAB]) OR (Nociplastic Low Back Pain[TIAB]) OR (Nociplastic Pain[TIAB]) OR (Non-specific Low Back Pain[TIAB])) AND ((Exercise Therapy[MH]) OR (Exercise Therap*[TIAB]) OR (Rehabilitation Exercise*[TIAB]) OR (Resistance Training[TIAB]) OR (Strength Training[TIAB]) OR (Weight-Lifting Exercise Program*[TIAB]) OR (Endurance Training[TIAB]) OR (Aquatic Therap*[TIAB]) OR (Pool Therap*[TIAB]) OR (Sensorimotor Exercise[TIAB]) OR (Sensorimotor Training[TIAB]) OR (Core Balance[TIAB]) OR (Core Stability[TIAB]) OR (Exercise Movement Technique*[TIAB]) OR (Pilates Training[TIAB]) OR (Yoga[TIAB]) OR (Tai Ji[TIAB]) OR (Tai Chi[TIAB]) OR (Muscle Stretching Exercise*[TIAB]) OR (Proprioceptive Neuromuscular Facilitation[TIAB]) OR (PNF Stretching[TIAB]) OR (Breathing Exercise*[TIAB]) OR (Respiratory Muscle Training[TIAB]) OR (McKenzie Method[TIAB])) AND ((Musculoskeletal Manipulations[MH]) OR (Musculoskeletal Manipulation*[TIAB]) OR (Craniosacral Massage[TIAB]) OR (Reflexology[TIAB]) OR (Manipulation Therap*[TIAB]) OR (Manual Therap*[TIAB]) OR (Manipulation, Osteopathic[MH] OR (Manipulation, Osteopathic[TIAB]) OR (Osteopathic Manipulative Treatment*[TIAB]) OR (Chiropractic Manipulation[TIAB]) OR (Chiropractic Spinal Adjustment*[TIAB]) OR (Neurodynamic Mobilization*[TIAB]) OR (Neurodynamic Technique*[TIAB]) OR (Mulligan Mobilization[TIAB]) OR (Mobilization With Movement[TIAB]) OR (Cyriax Friction Massage[TIAB]) OR (Myofascial Release Therap*[TIAB]) OR (Maitland Mobilization[TIAB])) AND ((Musculoskeletal Pain[MH]) OR (Quality of Life[MH]) OR (Disability[Tiab]) OR (Sleep Quality[MH])) AND ((Clinical Trial[TIAB]) OR (Controlled Trial[TIAB]) OR (Randomized Controlled Trial[TIAB]))	282	3
Web of Science	*Low Back Pain* OR *Chronic Low Back Pain* OR *Nociplastic Low Back Pain* OR *Nociplastic Pain* OR *Non-specific Low Back Pain* (Topic) AND *Exercise Therapy* OR *Rehabilitation Exercise* OR *Resistance Training* OR *Strength Training* OR *Weight-Lifting Exercise Program* OR *Endurance Training* OR *Aquatic Therapy* OR *Sensorimotor Exercise* OR * Sensorimotor Training* OR *Core Balance* OR *Core Stability* OR *Exercise Movement Techniques* OR *Pilates Training* OR *Yoga* OR *Tai Chi* OR *Muscle	421	1

	Stretching Exercise* OR *PNF Stretching* OR *Breathing Exercise* OR *Respiratory Muscle Training* OR *McKenzie Method* (Topic) AND *Musculoskeletal Manipulations* OR *Craniosacral Massage* OR *Reflexology* OR *Manipulation Therapy* OR *Manual Therapy* OR *Osteopathic Manipulative Treatment* OR *Chiropractic Manipulation* OR *Neurodynamic Mobilization* OR *Neurodynamic Technique* OR *Mulligan Mobilization* OR *Mobilization With Movement* OR *Cyriax Friction Massage* OR *Myofascial Release Therapy* OR *Maitland Mobilization* (Topic) AND *Musculoskeletal Pain* OR *Quality of Life* OR *Disability* OR *Sleep Quality* (Topic) AND *Clinical Trial* OR *Controlled Trial* OR *Randomized Controlled Trial* (Topic)		
Scopus	(TITLE-ABS-KEY ("Low Back Pain" OR "Chronic Low Back Pain" OR "Nociplastic Low Back Pain" OR "Nociplastic Pain" OR "Non-specific Low Back Pain") AND TITLE-ABS-KEY ("Exercise Therapy" OR "Rehabilitation Exercise" OR "Resistance Training" OR "Strength Training" OR "Weight-Lifting Exercise Program" OR "Endurance Training" OR "Aquatic Therapy" OR "Sensorimotor Exercise" OR "Sensorimotor Training" OR "Core Balance" OR "Core Stability" OR "Exercise Movement Techniques" OR "Pilates Training" OR "Yoga" OR "Tai Chi" OR "Muscle Stretching Exercise" OR "PNF Stretching" OR "Breathing Exercise" OR "Respiratory Muscle Training" OR "McKenzie Method") AND TITLE-ABS-KEY ("Musculoskeletal Manipulations" OR "Craniosacral Massage" OR "Reflexology" OR "Manipulation Therapy" OR "Manual Therapy" OR "Osteopathic Manipulative Treatment" OR "Chiropractic Manipulation" OR "Neurodynamic Mobilization" OR "Neurodynamic Technique" OR "Mulligan Mobilization" OR "Mobilization With Movement" OR "Cyriax Friction Massage" OR "Myofascial Release Therapy" OR "Maitland Mobilization") AND TITLE-ABS-KEY ("Musculoskeletal Pain" OR "Quality of Life" OR "Disability" OR "Sleep Quality") AND TITLE-ABS-KEY ("Clinical Trial" OR "Controlled Trial" OR "Randomized Controlled Trial"))	116	1
PEDro	Chronic Low Back Pain AND Musculoskeletal Manipulations AND Exercise Therapy<br>Chronic Low Back Pain AND massage AND Therapeutic Exercise<br>Low Back Pain AND Manual Therapy AND Exercise therapy	179	0
Google Scholar	Manual Therapy AND Exercise therapy AND Manual Therapy AND Chronic Low Back Pain	1	0
	**Total articles**	1510	6

Table S2. Evidence synthesis of outcomes based on certainty and importance using the GRADE method for pain intensity, disability, and physical function.

**Certainty assessment**								**Patients**		**Effect**		**Certainty**	**Importance**
	**Studies**	**Design**	**Risk of bias**	**Inconsistency**	**Indirect evidence**	**Imprecision**	**Others**	**Exercise Therapy**	**Manual Therapy**	**Relative (95% CI)**	**Absolute (95% CI)**		
Pain intensity	6	ECA	very serious	very serious	not serious	extremely serious	none	465	278	-	SMD 0,14 (-0.02 to 0.3)	⊕◯◯◯ very low	2/11
Disability	5	ECA	very serious	very serious	not serious	extremely serious	none	456	270	-	SMD -0,04 (-0,19 to 0,12)	⊕◯◯◯ very low	2/11
Physical function	2	ECA	very serious	very serious	not serious	extremely serious	none			-	SMD -0.05 (-0.18 to 0.08)	⊕◯◯◯ very low	2/11

Table S3. Sensitivity analysis to assess the impact of methodology and characteristics of included studies on the outcome of pain intensity (SMD).

**Excluded trial**	**Temporality**	**SMD (CI 95%)**	***P* for SMD**	**I^2**	***P* for heterogeneity**
Ferreira et al., 2010 (A)	***0-3 months***	0.33 (-0.03, 0.69)	0.07	79%	<0.0001
	Total	0.14 (-0.02, 0.30)	0.09	62%	0.0006
Ferreira et al., 2010 (B)	***0-3 months***	0.35 (-0.01, 0.71)	0.05	78%	<0.0001
	Total	0.15 (-0.02, 0.31)	0.08	61%	0.0007
Ferreira et al., 2007 (A)	***0-3 months***	0.33 (-0.07, 0.73)	0.10	79%	<0.0001
	Total	0.15 (-0.04, 0.30)	0.13	61%	0.0007
Ferreira et al., 2007 (B)	***0-3 months***	0.38 (0.00, 0.76)	0.05	76%	0.0001
	Total	0.16 (-0.01, 0.33)	0.07	60%	0.0009
Zhang et al., 2022	***0-3 months***	0.29 (-0.07, 0.65)	0.11	78%	<0.0001
	Total	0.13 (-0.03, 0.29)	0.12	61%	0.0008
Oliveira et al., 2020	***0-3 months***	0.14 (-0.07, 0.34)	0.19	34%	0.16
	Total	0.08 (-0.02, 0.17)	0.14	2%	0.43
Bronfort et al., 2011 (A)	***0-3 months***	0.36 (-0.05, 0.76)	0.08	78%	<0.0001
	Total	0.15 (-0.03, 0.32)	0.76	0%	0.99
Bronfort et al., 2011 (B)	***0-3 months***	0.38 (-0.01, 0.77)	0.06	76%	0.0001
	Total	0.16 (-0.02, 0.33)	0.07	61%	0.0009
Ulger et al., 2017	***0-3 months***	0.26 (-0.11, 0.63)	0.16	74%	0.0003
	Total	0.10 (-0.06, 0.25)	0.22	53%	0.007
Ferreira et al., 2007 (A)	***3-6 months***	0.10 (-0.14, 0.34)	0.41	26%	0.26
	Total	0.14 (-0.03, 0.31)	0.10	62%	0.0006
Ferreira et al., 2007 (B)	***3-6 months***	0.15 (-0.06, 0.35)	0.15	0%	0.39
	Total	0.15 (-0.02, 0.32)	0.08	61%	0.0008

Bronfort et al., 2011 (A)	***3-6 months***	0.14 (-0.08, 0.36)	0.21	10%	0.33
	Total	0.15 (-0.02, 0.32)	0.09	61%	0.0007
Bronfort et al., 2011 (B)	***3-6 months***	0.02 (-0.19, 0.23)	0.88	0%	0.84
	Total	0.12 (-0.05, 0.29)	0.15	60%	0.001
Ferreira et al., 2007 (A)	***>6 months***	-0.04 (-0.24, 0.17)	0.72	0%	0.96
	Total	0.15 (-0.02, 0.32)	0.09	61%	0.0007
Ferreira et al., 2007 (B)	***>6 months***	-0.02 (-0.23, 0.18)	0.83	0%	0.96
	Total	0.15 (-0.02, 0.32)	0.08	61%	0.0008
Bronfort et al., 2011 (A)	***>6 months***	-0.02 (-0.23, 0.20)	0.89	0%	0.98
	Total	0.15 (-0.02, 0.33)	0.08	61%	0.0009
Bronfort et al., 2011 (B)	***>6 months***	-0.04 (-0.25,0.17)	0.71	0%	0.97
	Total	0.15 (-0.02, 0.32)	0.09	61%	0.0007

SMD: standardized mean difference; CI: confidence Interval

Table S4. Sensitivity analysis to assess the impact of methodology and characteristics of included studies on the outcome of disability (SMD).

**Excluded trial**	**Temporality**	**SMD (CI 95%)**	***P* for SMD**	**I^2**	***P* for heterogeneity**
Ferreira et al., 2010 (A)	***0-3 months***	0.19 (-0.12, 0.50)	0.22	72%	0.002
	Total	-0.03 (-0.19, 0.12)	0.007	0%	0.76
Ferreira et al., 2010 (B)	***0-3 months***	0.15 (-0.16, 0.47)	0.34	72%	0.001
	Total	-0.05 (-0.20, 0.11)	0.56	59%	0.002
Ferreira et al., 2007 (A)	***0-3 months***	0.20 (-0.15, 0.55)	0.26	72%	0.001
	Total	-0.04 (-0.21, -0.07)	0.63	59%	0.002
Ferreira et al., 2007 (B)	***0-3 months***	0.26 (-0.03, 0.54)	0.08	59%	0.02
	Total	-0.01 (-0.17, 0.14)	0.86	56%	0.004
Oliveira et al., 2020	***0-3 months***	0.06 (-0.19, 0.32)	0.62	55%	0.04
	Total	-0.08 (-0.21, 0.05)	0.22	40%	0.06
Bronfort et al., 2011 (A)	***0-3 months***	0.19 (-0.17, 0.55)	0.30	72%	0.001
	Total	-0.05 (-0.21, 0.12)	0.59	59%	0.002
Bronfort et al., 2011 (B)	***0-3 months***	0.21 (-0.14, 0.56)	0.24	71%	0.002
	Total	-0.04 (-0.20,0.13)	0.67	60%	0.002
Ulger et al., 2017	***0-3 months***	0.08 (-0.21, 0.37)	0.59	59%	0.02
	Total	-0.09 (-0.23, 0.04)	0.16	38%	0.07

Ferreira et al., 2007 (A)	***3-6 months***	-0.18 (-0.39, 0.02)	0.08	0%	0.99
	Total	-0.06 (-0.21, 0.11)	0.55	58%	0.002
Ferreira et al., 2007 (B)	***3-6 months***	-0.09 (-0.29, 0.12)	0.40	0%	0.37
	Total	-0.02 (-0.19, 0.14)	0.77	59%	0.002
Bronfort et al., 2011 (A)	***3-6 months***	-0.08 (-0.29, 0.13)	0.46	0%	0.39
	Total	-0.02 (-0.19, 0.14)	0.78	59%	0.002
Bronfort et al., 2011 (B)	***3-6 months***	-0.09 (-0.31, 0.13)	0.41	4%	0.35
	Total	-0.03 (-0.19, 0.14)	0.76	59%	0.002
Ferreira et al., 2007 (A)	***>6 months***	-0.24 (-0.44, -0.04)	0.02	0%	0.56
	Total	-0.02 (-0.18, 0.14)	0.81	58%	0.002
Ferreira et al., 2007 (B)	***>6 months***	-0.20 (-0.40, 0.01)	0.06	0%	0.92
	Total	-0.01 (-0.17, 0.15)	0.89	55%	0.006
Bronfort et al., 2011 (A)	***>6 months***	-0.28 (-0.49, -0.07)	0.010	00%	0.66
	Total	-0.03 (-0.19, 0.14)	0.76	59%	0.002
Bronfort et al., 2011 (B)	***>6 months***	-0.27 (-0.48, -0.06)	0.01	0%	0.62
	Total	-0.02 (-0.19, 0.14)	0.77	59%	0.002

SMD: standardized mean difference; CI: confidence Interval

Table S5. Sensitivity analysis to assess the impact of methodology and characteristics of included studies on the outcome of physical function (SMD).

Excluded trial	Temporality	SMD (CI 95%)	***P*** for SMD	I^2	***P*** for heterogeneity
Bronfort et al., 2011 (A)	***0-3 months***	0.10 (-0.15, 0.35)	0.44	81%	0.02
	Total	-0.06 (-0.20, 0.08)	0.41	35%	0.17
Bronfort et al., 2011 (B)	***0-3 months***	0.18 (-0.07,0.80)	0.15	66%	0.09
	Total	-0.03 (-0.17, 0.11)	0.65	31%	0.20
Ulger et al., 2017	***0-3 months***	-0.09 (-0.33, 0.15)	0.44	0%	0.53
	Total	-0.12 (-0.26, 0.02)	0.09	0%	0.99
Bronfort et al., 2011 (A)	***3-6 months***				
	Total	-0.04 (-0.18, 0.10)	0.59	33%	0.19
Bronfort et al., 2011 (B)	***3-6 months***				
	Total	-0.04 (-0.18, 0.10)	0.55	34%	0.18
Bronfort et al., 2011 (A)	***>6 months***				
	Total	-0.03 (-0.17, 0.11)	0.67	29%	0.21
Bronfort et al., 2011 (B)	***>6 months***				
	Total	-0.05 (-0.19, 0.09)	0.51	35%	0.17

SMD: standardized mean difference; CI: confidence interval

Table S6. Sensitivity analysis to assess the impact of methodology and characteristics of included studies on the outcome of pain intensity (MD).

Excluded trial	Temporality	MD (CI 95%)	*P* for MD	I^2	*P* for heterogeneity
Ferreira et al., 2010 (A)	*0-3 months*	0.58 (-0.06, 1.22)	0.07	85%	<0.00001
	Total	0.28 (-0.01, 0.57)	0.06	73%	<0.00001
Ferreira et al., 2010 (B)	*0-3 months*	0.62 (-0.01, 1.26)	0.06	85%	<0.00001
	Total	0.29 (0.00, 0.58)	0.05	73%	<0.00001
Ferreira et al., 2007 (A)	*0-3 months*	0.57 (-0.12, 1.26)	0.11	85%	<0.00001
	Total	0.27 (-0.03, 0.57)	0.08	73%	<0.00001
Ferreira et al., 2007 (B)	*0-3 months*	0.65 (-0.02, 1.32)	0.06	84%	<0.00001
	Total	0.30 (0.01, 0.60)	0.05	73%	<0.00001
Zhang et al., 2022	*0-3 months*	0.52 (-0.12, 1.17)	0.11	85%	<0.00001
	Total	0.26 (-0.04, 0.55)	0.09	73%	<0.00001
Oliveira et al., 2020	*0-3 months*	0.22 (-0.11, 0.55)	0.18	39%	0.12
	Total	0.13 (-0.03, 0.29)	0.12	15%	0.28
Bronfort et al., 2011 (A)	*0-3 months*	0.62 (-0.12, 1.35)	0.10	84%	<0.00001
	Total	0.29 (-0.02, 0.60)	0.72	0%	0.99
Bronfort et al., 2011 (B)	*0-3 months*	0.65 (-0.05, 1.35)	0.07	83%	<0.00001
	Total	0.31 (0.00, 0,61)	0.05	73%	<0.00001
Ulger et al., 2017	*0-3 months*	0.48 (-0.19, 1.15)	0.16	83%	<0.00001
	Total	0.22 (-0.07, 0.51)	0.13	70%	<0.0001
Ferreira et al., 2007 (A)	*3-6 months*	0.18 (-0.20, 0.56)	0.36	47%	0.15
	Total	0.28 (-0.02, 0.58)	0.07	73%	<0.00001
Ferreira et al., 2007 (B)	*3-6 months*	0.24 (-0.10, 0.57)	0.16	33%	0.22
	Total	0.30 (0.00, 0.60)	0.05	73%	<0.00001
Bronfort et al., 2011 (A)	*3-6 months*	0.31 (-0.04, 0.65)	0.08	10%	0.33
	Total	0.30 (-0.01, 0.61)	0.06	73%	<0.00001
Bronfort et al., 2011 (B)	*3-6 months*	0.02 (-0.29, 0.33)	0.90	0%	0.83
	Total	0.26 (-0.05, 0.57)	0.10	72%	<0.00001
Ferreira et al., 2007 (A)	*>6 months*	-0.06 (-0.35, 0.23)	0.70	0%	0.95
	Total	0.29 (-0.01, 0.59)	0.06	73%	<0.00001
Ferreira et al., 2007 (B)	*>6 months*	-0.04 (-0.33, 0.25)	0.78	0%	0.95

	Total	0.30 (0.00, 0.60)	0.05	73%	<0.00001
Bronfort et al., 2011 (A)	***>6 months***	-0.02 (-0.36, 0.32)	0.91	0%	0.97
	Total	0.31 (0.00, 0.61)	0.05	72%	<0.00001
Bronfort et al., 2011 (B)	***>6 months***	-0.08 (-0.42, 0.26)	0.65	0%	0.97
	Total	0.30 (-0.01, 0.61)	0.06	73%	<0.00001

MD: mean difference; CI: confidence Interval

Table S7. Sensitivity analysis to assess the impact of methodology and characteristics of included studies on the outcome of physical function (MD).

**Excluded trial**	**Temporality**	**MD (CI 95%)**	***P* for MD**	**I^2**	***P* for heterogeneity**
**Bronfort et al., 2011 (A)**	**0-3 months**	2.01 (-4.70, 8.72)	0.56	84%	0.01
	**Total**	-0.64 (-1.50, 0.22)	0.15	28%	0.23
**Bronfort et al., 2011 (B)**	**0-3 months**	2.39 (-3.40, 8.19)	0.42	79%	0.03
	**Total**	-0.46 (-1.33, 0.41)	0.30	29%	0.22
**Ulger et al., 2017**	**0-3 months**	-0.55 (-1.68, 0.58)	0.34	0%	0.44
	**Total**	-0.70 (-1.36. -0.04)	0.04	0%	0.96
**Bronfort et al., 2011 (A)**	**3-6 months**				
	**Total**	-0.49 (-1.37, 0.39)	0.27	31%	0.20
**Bronfort et al., 2011 (B)**	**3-6 months**				
	**Total**	-0.51 (-1.40, 0.38)	0.26	32%	0.20
**Bronfort et al., 2011 (A)**	**>6 months**				
	**Total**	-0.44 (-1.30, 0.42)	0.31	27%	0.23
**Bronfort et al., 2011 (B)**	**>6 months**				
	**Total**	-0.55 (-1.44, 0.34)	0.23	32%	0.20

MD: mean difference; CI: confidence interval

Table S8. Sensitivity analysis to assess the impact of methodology and characteristics of included studies on the outcome of pain intensity, excluding Oliveira et al. and Bronfort et al. (SMD)

Excluded trial	Temporality	SMD (CI 95%)	*P* for SMD	I^2	*P* for heterogeneity
Ferreira et al., 2010 (A)	***0-3 months***	0.23 (0.02, 0.44)	0.03	51%	0.09
	Total	0.11 (-0.03, 0.25)	0.14	29%	0.19
Ferreira et al., 2010 (B)	***0-3 months***	0.25 (0.04, 0.46)	0.02	46%	0.12
	Total	0.11 (-0.03, 0.25)	0.12	26%	0.22
Ferreira et al., 2007 (A)	***0-3 months***	0.23 (-0.01, 0.47)	0.07	52%	0.08
	Total	0.09 (-0.06. 024)	0.25	26%	0.21
Ferreira et al., 2007 (B)	***0-3 months***	0.36 (0.12, 0.60)	0.003	2%	0.40
	Total	0.14 (-0.01, 0.29)	0.07	18%	0.28
Zhang et al., 2022	***0-3 months***	0.21 (0.00, 0.42)	0.05	49%	0.10
	Total	0.10 (-0.04, 0.24)	0.18	23%	0.24
Ulger et al., 2017	***0-3 months***	0.07 (-0.17, 0.31)	0.58	0%	0.59
	Total	0.03 (-0.12, 0.18)	0.72	0%	0.90
Ferreira et al., 2007 (A)	***3-6 months***				
	Total	0.11 (-0.04, 0.25)	0.17	29%	0.19
Ferreira et al., 2007 (B)	***3-6 months***				
	Total	0.13 (-0.02, 0.28)	0.09	23%	0.23
Ferreira et al., 2007 (A)	***>6 months***				
	Total	0.12 (-0.03, 0.27)	0.11	26%	0.21
Ferreira et al., 2007 (B)	***>6 months***				
	Total	0.13 (-0.02, 0.28)	0.09	24%	0.23

SMD: standardized mean difference; CI: confidence interval

Table S9. Sensitivity analysis to assess the impact of methodology and characteristics of included studies on the outcome of disability, excluding Oliveira et al. and Bronfort et al. (SMD)

Excluded trial	Temporality	SMD (CI 95%)	*P* for SMD	I^2	*P* for heterogeneity
Ferreira et al., 2010 (A)	***0-3 months***	0.13 (-0.35, 0.60)	0.60	76%	0.006
	Total	-0.05 (-0.30, 0.21)	0.72	66%	0.004
Ferreira et al., 2010 (B)	***0-3 months***	0.04 (-0.43, 0.51)	0.86	76%	0.006
	Total	-0.08 (-0.32, 0.17)	0.55	65%	0.005
Ferreira et al., 2007 (A)	***0-3 months***	0.11 (-0.50, 0.71)	0.73	77%	0.005
	Total	-0.06 (-0.34, 0.21)	0.22	66%	0.004
Ferreira et al., 2007 (B)	***0-3 months***	0.26 (-0.12, 0.64)	0.19	44%	0.15
	Total	-0.01 (-0.26, 0.25)	0.96	61%	0.01
Ulger et al., 2017	***0-3 months***	-0.13 (-0.40, 0.14)	0.33	6%	0.36
	Total	-0.16 (-0.32, 0.00)	0.05	9%	0.36
Ferreira et al., 2007 (A)	***3-6 months***			%	
	Total	-0.08 (-0.35, 0.19)	0.55	64%	0.006
Ferreira et al., 2007 (B)	***3-6 months***			%	
	Total	-0.03 (-0.31. 0.24)	0.82	66%	0.005
Ferreira et al., 2007 (A)	***>6 months***			%	
	Total	-0.02 (-0.29,0.25)	0.87	64%	0.006
Ferreira et al., 2007 (B)	***>6 months***			%	
	Total	0.00 (-0.25, 0.25)	1.00	59%	0.02

SMD: standardized mean difference; CI: confidence interval

Table S10. Sensitivity analysis to assess the impact of methodology and characteristics of included studies on the outcome of pain intensity, excluding Oliveira et al. and Bronfort et al. (MD)

Excluded trial	Temporality	MD (CI 95%)	*P* for MD	I^2	*P* for heterogeneity
**Ferreira et al., 2010 (A)**	**0-3 months**	0.44 (0.08, 0.79)	0.02	49%	0.10
	**Total**	0.23 (-0.03, 0.48)	0.08	28%	0.19
**Ferreira et al., 2010 (B)**	**0-3 months**	0.47 (0.11, 0.82)	0.01	43%	0.14
	**Total**	0.24 (-0.02, 0.49)	0.07	25%	0.23
**Ferreira et al., 2007 (A)**	**0-3 months**	0.43 (0.03,0.83)	0.04	50%	0.09
	**Total**	0.20 (-0.07, 0.46)	0.15	26%	0.21
**Ferreira et al., 2007 (B)**	**0-3 months**	0.63 (0.23, 1.03)	0.002	0%	0.41
	**Total**	0.28 (0.02, 0.55)	0.04	16%	0.30
**Zhang et al., 2022**	**0-3 months**	0.39 (0.03, 0.75)	0.03	48%	0.10
	**Total**	0.20 (-0.06, 0.45)	0.13	23%	0.24
**Ulger et al., 2017**	**0-3 months**	0.14 (-0.28, 0.57)	0.51	0%	0.58
	**Total**	0.06 (-0.21, 0.34)	0.66	0%	0.89
**Ferreira et al., 2007 (A)**	**3-6 months**				
	**Total**	0.23 (-0.04, 0.59)	0.10	28%	0.19
**Ferreira et al., 2007 (B)**	**3-6 months**				
	**Total**	0.27 (0.00, 0.54)	0.05	22%	0.25
**Ferreira et al., 2007 (A)**	**>6 months**				
	**Total**	0.25 (-0.02, 0.52)	0.07	26%	0.21
**Ferreira et al., 2007 (B)**	**>6 months**				
	**Total**	0.26 (0.00, 0.53)	0.05	23%	0.24

MD: mean difference; CI: confidence Interval
